# Multi‐Omics Analysis Reveals Causal Relationships and Potential Mediators Between Dietary Preferences and Risk of NAFLD

**DOI:** 10.1002/fsn3.70446

**Published:** 2025-06-23

**Authors:** Qingan Fu, Jierui Liu, Zhekang Liu, Tianzhou Shen, Qingyun Yu, Huangxin Zhu, Shisheng Wu, Rixiang Liu, Deju Zhang, Xiao Liu, Xiaoping Yin, Jianping Liu, Yanze Wu, Jing Zhang, Peng Yu

**Affiliations:** ^1^ Department of Endocrinology and Metabolism, the Second Affiliated Hospital, Jiangxi Medical College Nanchang University Nanchang China; ^2^ Cardiovascular Medicine Department The Second Affiliated Hospital of Nanchang University Nanchang China; ^3^ Gastroenterology Department The Second Affiliated Hospital of Nanchang University Nanchang China; ^4^ Rheumatology and Immunology Department The Second Affiliated Hospital of Nanchang University Nanchang China; ^5^ Food and Nutritional Sciences, School of Biological Sciences The University of Hong Kong Pokfulam Hong Kong; ^6^ Department of Cardiology, Sun Yat‐Sen Memorial Hospital Sun Yat‐Sen University Guangzhou China; ^7^ Department of Neurology Affiliated Hospital of Jiujiang University Jiujiang China; ^8^ Department of Neurosurgery, the Second Affiliated Hospital Jiangxi Medical College, Nanchang University Nanchang China; ^9^ Department of Anesthesiology, the Second Affiliated Hospital, Jiangxi Medical College Nanchang University Nanchang China

**Keywords:** dietary preferences, FTO gene, inflammatory factors, Mendelian randomization, non‐alcoholic fatty liver disease

## Abstract

Non‐alcoholic fatty liver disease (NAFLD) is a prevalent condition closely associated with obesity and metabolic syndrome, with its global incidence on the rise. This study aims to explore the causal relationship between dietary preferences and NAFLD risk using multi‐omics analysis, and to comprehensively explore possible mediating factors and their underlying mechanisms. We analyzed data from genome‐wide association studies (GWAS) to assess the potential genetic links between various dietary preferences and NAFLD. A two‐step Mendelian randomization (MR) analysis was conducted to evaluate whether dietary preferences affect NAFLD risk by regulating inflammatory factors. Further, co‐localization analysis was used to identify gene loci driving the causal relationships between dietary preferences and NAFLD risk. Finally, clinical cross‐sectional data from the National Health and Nutrition Examination Survey (NHANES) and bioinformatics analysis were used to validate the findings.MR analysis revealed that a preference for a low‐calorie diet significantly reduces NAFLD risk by modulating DNER. Co‐localization analysis identified the FTO gene variant rs28429148 as a key driver of the causal relationship between soft cheese and fruit juice preferences, with soft cheese increasing and fruit juice reducing NAFLD risk. These findings were further validated by clinical cross‐sectional and bioinformatics analysis. This study, for the first time, comprehensively elucidates the causal relationship between dietary preferences and NAFLD risk from a multi‐omics perspective and identifies FTO and DNER as potential therapeutic targets. These findings provide new insights into the importance of personalized dietary interventions in the prevention of NAFLD and informs clinical treatment.

## Graphical abstract

This study combines Mendelian randomization, co‐localization, and clinical cross‐sectional analyses to reveal that preferences for fruit juice and soft cheese have opposite causal effects on NAFLD risk, mediated by the FTO gene. Moreover, low‐calorie dietary preferences reduce NAFLD risk through up‐regulation of inflammatory protein DNER. These findings suggest that genetic determinants of dietary preferences may inform precision nutrition strategies and identify FTO and DNER as potential therapeutic targets in NAFLD management.

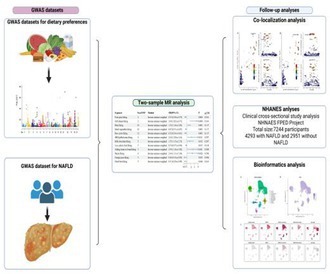

## 1 Introduction

Non‐alcoholic fatty liver disease (NAFLD) has become the most common liver disease in the world. It is characterized by abnormal deposition of non‐alcoholic fat in the liver and affects more than a quarter of the world's population (Lazo et al. 2013). The increasing incidence of NAFLD is attributed to increases in obesity and metabolic syndrome. The continuous progression of NAFLD can gradually transform it into non‐alcoholic steatohepatitis (NASH), liver fibrosis and even liver cancer. In the United States, the direct treatment costs for NAFLD patients exceed $100 billion each year, which places a heavy medical burden on patients and society (Younossi et al. 2016). In recent years, as researchers have studied the pathogenesis of NAFLD, many intervention and treatment measures have been proposed. This year, Resmetirom (Rezdiffra), a drug targeting NASH, was preliminarily approved by the United States Food and Drug Administration (FDA), but its long‐term effectiveness and safety still need further study (Harrison et al. 2024; Sookoian and Pirola 2024). The treatment of NAFLD is still unclear, and lifestyle interventions such as physical exercise and a healthy diet are still considered the most effective strategies for early intervention in the occurrence or progression of NAFLD, in particular, healthy dietary patterns have demonstrated significant NAFLD protective effects (Chen, Fan, et al. 2023).

Researchers have shown that lifestyle dietary patterns play an extremely important role in the reversal or promotion of NAFLD in its early stages, with Western dietary patterns promoting the development and progression of NAFLD, whereas low‐fat diets, ketogenic diets, and the Mediterranean dietary pattern are generally recognized as being able to reduce the risk of NAFLD or even treat NAFLD (Quetglas‐Llabrés et al. 2023; European Association for the Study of the Liver (EASL) et al. 2016). This may be because different dietary patterns have different regulatory effects on the inflammatory response in patients with NAFLD, and dietary intake can change the risk of NAFLD through changes in inflammatory protein levels (Quetglas‐Llabrés et al. 2024). Nevertheless, the current research on diet and NAFLD is characterized by small sample sizes and a small number of food groups, and a comprehensive study is urgently needed to explore the correlation between different dietary intakes and NAFLD risk from a multidimensional perspective, as well as the targets and mechanisms of action.

A recent study suggested that differences in food intake due to dietary preferences among different individuals may be genetically linked, suggesting that we can explore the associations and mechanisms between dietary preferences and fatty liver from the perspective of genetic variation (May‐Wilson et al. 2022). Mendelian randomization (MR) is an epidemiological method of causal inference based on the random assignment of genetic variants, which uses instrumental variables (IVs) instead of exposures and endpoints to infer causality, and avoids the drawbacks of small sample sizes and the susceptibility to confounding and reverse causality in traditional clinical studies (Fu et al. 2023; Liu, Fu, Yu, et al. 2024). The aim of this study was to combine two‐sample MR (TSMR) and mediated MR, co‐localization analyses, bioinformatics analyses, and clinical cross‐sectional studies to explore the associations, contributing factors, and specific mechanisms between dietary preferences and the risk of NAFLD and to provide new intervention modalities and therapeutic targets for the risk management of NAFLD.

## 2 Method

### 2.1 Study Design

**FIGURE 1:**
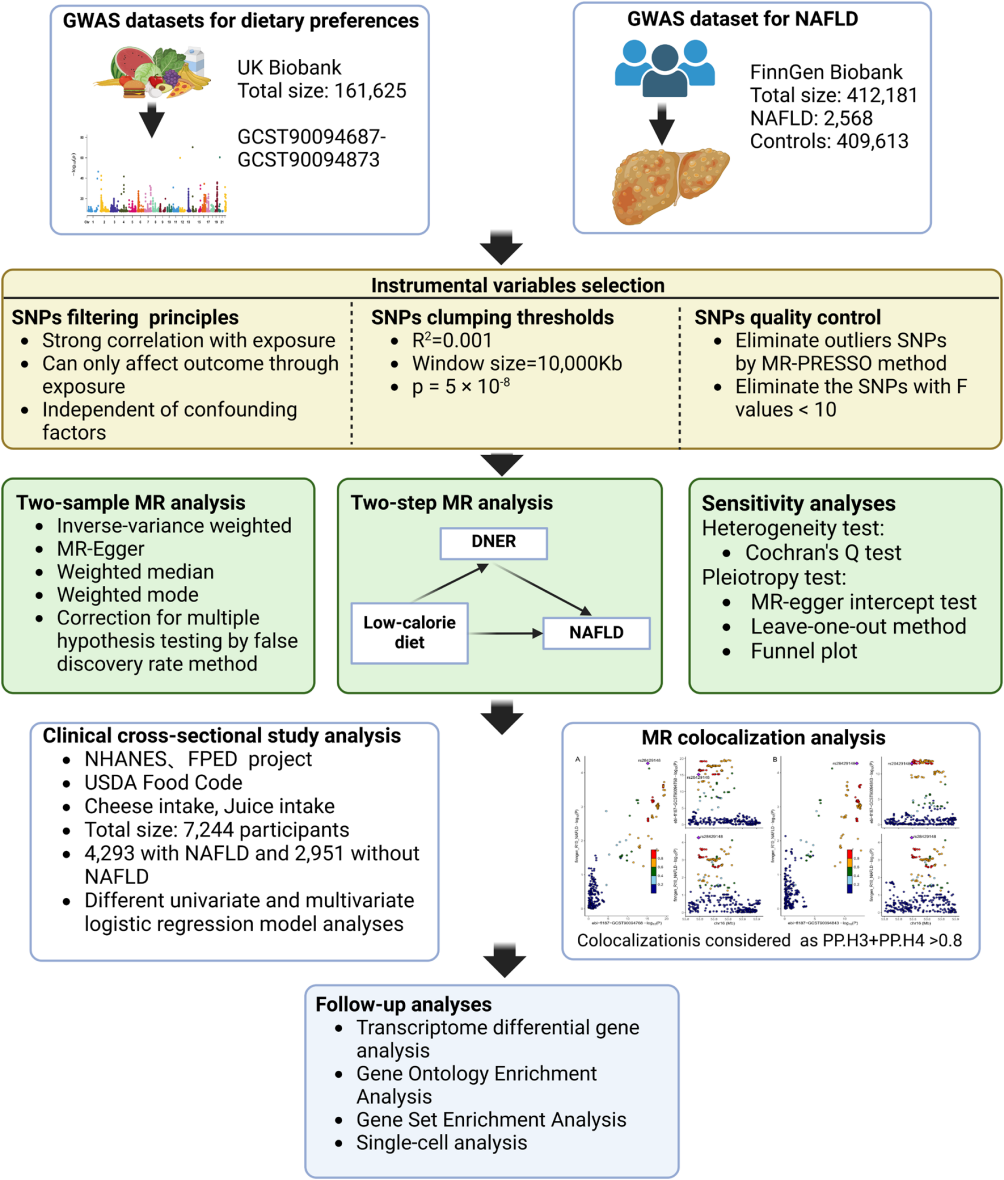
Design and flowchart of this study. DNER = delta and notch‐like epidermal growth factor‐related receptor, FPED = Nutritional Dietary Research Database, GWAS = Genome‐Wide Association Study, MR = Mendelian randomization, MR‐PRESSO = Mendelian Randomization Pleiotropy RESidual Sum and Outlier, NAFLD = non‐alcoholic fatty liver disease, NHANES = National Health and Nutrition Examination Survey, SNP = single nucleotide polymorphism, UK = United Kingdom.

In this study, we first used TSMR to explore the possible causal relationship between various dietary liking and NAFLD risk, and further conducted two‐step MR analysis on the significantly correlated causal relationships obtained from the analysis to explore the mediating factors that may mediate the causal relationship. Secondly, the results obtained from the TSMR analysis were corrected for multiple hypothesis testing, and the exposure and outcome that were still significant after correction were co‐localized to confirm the gene variant targets that drive the common causal relationship (Fu et al. 2023). TSMR analysis needs to meet three basic assumptions: first, the IVs used must be closely related to the exposure phenotype; second, the IVs cannot be associated with common confounding factors; third, the IVs can only affect the outcome phenotype through the exposure phenotype (Davies et al. 2018; Pan et al. 2025). The design of all MR‐related analyses strictly follows the “Strengthening the Reporting of Observational Studies in Epidemiology Using Mendelian Randomization” (STROBE‐MR) guidelines, and the complete STROBE‐MR list can be found in the Supporting Informations (Skrivankova et al. 2021; Liu, Fu, Shao, et al. 2024). In addition, this study conducted a clinical research analysis based on the National Health and Nutrition Examination Survey (NHANES) database to further verify the association between food intake caused by dietary preferences and the risk of NAFLD and improve the reliability of the conclusions. Finally, we conducted multi‐omics analysis based on the co‐localized targets found in the co‐localized MR analysis, and used various bioinformatics methods such as Gene Ontology (GO) enrichment, Gene Set Enrichment Analysis (GSEA), and single‐cell analysis to deeply explore the expression and mechanism of TSMR co‐localized targets in NAFLD patients. All the procedures of this study are briefly shown in Figure 1.

### 2.2 Data Sources

#### 2.2.1 GWAS Datasets

The dietary preferences GWAS dataset comes from 161,625 participants in the UK Biobank, and researchers used 9 scales to assess their preferences for 139 specific foods. The study found that differences in dietary preferences may be driven by genetic factors, and ultimately determined a hierarchical map of food preferences for all participants and obtained 187 dietary preferences‐related phenotypic data for inclusion in our study (May‐Wilson et al. 2022). (ID: GCST90094687‐GCST90094873).

The GWAS dataset for NAFLD used in this study comes from the R10 cycle data of the FinnGen study. The total sample size of the dataset is 412,181, including 2568 NAFLD patients and 409,613 controls (Kurki et al. 2023). (ID: finngen_R10_NAFLD) The FinnGen study is a large‐scale genomics project (https://www.finngen.fi/en/access_results) that aims to link genetic variation with health in order to provide researchers with new evidence on disease mechanisms and susceptibility. The R10 cycle data analyzed a total of 412,181 participants (181,871 male and 230,310 female). The diagnosis of NAFLD patients in the dataset conforms to the International Classification of Diseases code (ICD‐10: K76).

The GWAS dataset of inflammatory proteins comes from a large‐scale study. The researchers recruited 14,824 participants from 11 cohorts, all of whom were targeted with Olink Target‐96 to measure GWAS data and inflammatory proteomics data. All data were sequenced in the Olink laboratory, and GWAS data of 91 inflammatory protein components were finally measured (Zhao et al. 2023). (ID: GCST90274758‐GCST90274848).

#### 2.2.2 GEO Dataset

The Series Matrix File data file of GSE260666 was downloaded from the GEO database (https://www.ncbi.nlm.nih.gov/gds) (Barrett et al. 2013), where GPL24676 is the license for the annotation platform. With 10 patients in the NAFLD group and 6 patients in the normal group, a total of 16 sets of transcriptome data of liver tissue were included (Luo et al. 2024). The GSE202379 data file was downloaded for single‐cell correlation analysis, where liver tissues were biopsied from 18 patients with different stages of NAFLD and 4 healthy donors (Gribben et al. 2024). Specific information about the dataset is available on the online website.

#### 2.2.3 Clinical Data

Clinical cross‐sectional data for this study came from the 2017–2020 NHANES program, which is led by the National Center for Health Statistics (NCHS), and Centers for Disease Control and Prevention (CDC) and is designed to help researchers assess the health and nutritional status of adults and children across the United States. The survey was approved by NCHS Research Ethics Review Board, and to ensure that participants' rights are protected, NHANES has ensured that all individuals participating in the study have signed an informed consent form and that all data are publicly available on the CDC or United States Department of Agriculture website (USDA) (Https://Wwwn.Cdc.Gov/Nchs/Nhanes/Continuousnhanes/Default.Aspx?Beginyear = 2017).

During the 2017–2020 cycle, NHANES staff evaluated participants for vibration‐controlled transient elastography (VCTE) using a FibroScan 502 Touch device to measure and record the coefficient of attenuation parameter (CAP) as an indicator of the degree of hepatic steatosis. In this study, we defined a CAP threshold of 248 dB/m, which is considered to be the optimal threshold for the diagnosis of hepatic steatosis, as the threshold for recognizing hepatic steatosis (Karlas et al. 2017; Liu, Fu, Su, et al. 2024). In addition, we excluded patients with excessive alcohol consumption (> 10 g/day in women and > 20 g/day in men) and other liver diseases (positive hepatitis B virus surface antigen or hepatitis C virus ribonucleic acid tests or with autoimmune hepatitis). A total of 7244 participants were included in the final study, including 4293 with NAFLD and 2951 without NAFLD.

Data on dietary intake in the NHANES program were collected by trained staff conducting 24‐h dietary recalls of all participants at the Mobile Examination Center (MEC) (Zhu et al. 2024). The 24‐h recalls recorded all foods and beverages consumed by each participant over a 24‐h period and numbered all food groups according to the USDA Food Code. The USDA Food and Nutritional Dietary Research Database (FPED) provided detailed information on the composition of each food item, including the amount of cheese and fruit juice, to calculate the specific total daily intake of cheese and fruit juice for all participants (Roark et al. 2022). All GWAS, GEO, and clinical data used in this study were obtained from published studies or public databases, and subject consent was obtained and reviewed by the ethics committee in the original study.

### 2.3 Statistical Analysis

#### 2.3.1 MR Analysis

When screening single nucleotide polymorphisms (SNPs) for inclusion as IVs in TSMR analysis, we set strict thresholds. SNPs need to meet the criteria of strong correlation with exposure phenotype (*p* < 5 × 10^−8^) and meet the criteria of *R* ^2^ = 0.001 and window size = 10,000 kb. In addition, to avoid the bias of weak IV and to ensure the validity of SNPs, we used Mendelian Randomization Pleiotropy RESidual Sum and Outlier (MR‐PRESSO) to remove outlier SNPs, and then excluded SNPs with F‐value less than 10 (Burgess and Thompson 2011). In TSMR analysis, the results of our IVW method were used as the main results (Burgess et al. 2015). However, considering that the IVW method may ignore blank IVs and heterogeneity, we additionally supplemented other MR methods to improve the reliability of the results, including MR‐Egger, weighted median and weighted mode (Bowden et al. 2015, 2016; Hartwig et al. 2017). In order to avoid possible false positive results in multi‐group data analysis in MR analysis, we applied false discovery rate (FDR) to correct multiple hypothesis testing for all results (Korthauer et al. 2019; Fu et al. 2024). In TSMR analysis, we considered *p* < 0.05 in the IVW method as a potential positive causal relationship, while *p* < 0.05 after FDR (*p_*FDR) correction was considered a strong positive and significant causal relationship.

To ensure the robustness of the results, we performed sensitivity analyses using multiple methods. Heterogeneity was assessed using the Cochrane Q test (Burgess et al. 2013), The leave‐one‐out method and MR‐egger intercept test and funnel plot were used to assess whether a single SNP affected the overall outcome and whether the outcome had horizontal pleiotropy (Verbanck et al. 2018).

#### 2.3.2 Two‐Step MR Analysis

We further performed two‐step MR based on the TSMR results, using dietary preferences and NAFLD with potential positive causal relationships in the MR results as exposures and outcomes, and 91 inflammatory proteins as potential mediators to assess whether inflammatory proteins mediated the causal relationship between dietary preferences and NAFLD. First, the effect of genetically determined dietary preferences on inflammatory proteins (β1) was calculated by two‐sample MR. Subsequently, the effect of inflammatory proteins on NAFLD (β2) was assessed. Finally, the indirect effect of each mediator was determined by dividing the product of the results of the two‐stage IVW method (β1 × β2) by the total effect.

#### 2.3.3 MR Colocalization Analysis

After TSMR analysis, we obtained significant causal relationships between various dietary preferences and NAFLD risk and performed FDR multiple correction. We used Bayesian colocalization analysis to explore gene targets that mediated strong positive causal relationships. Colocalization analysis is a method for estimating posterior probability, which is often used to assess whether the same genetic variation site jointly drives the causal effect between exposure and outcome phenotypes and mediates the causal relationship between the two. In this study, when the sum of PP.H3 + PP.H4 in the posterior probability of colocalization analysis is greater than 0.8, we believe that there is a colocalization target (Dunn et al. 2011). This means that within a certain gene region, the same causal variant affects both exposure and outcome. The larger the PP.H3 + PP.H4, the more likely the causal relationship between dietary preferences and NAFLD risk is driven by the common variation site. LocusCompare plots were used to display the results of colocalization analysis of different dietary preferences and NAFLD (Liu et al. 2019). All analyses related to TSMR were performed using the “TwoSampleMR,” “MRPRESSO,” and “coloc” R packages.

#### 2.3.4 Identification of Differentially Expressed Genes (DEGs)

The limma R package (Ritchie et al. 2015) was applied to compare the disease and normal groups. The screening cutoff criteria for DEGs were a *p* value < 0.05 and a |log fold change (FC)| > 0.5. The ggplot2 R package was used to generate volcano maps.

#### 2.3.5 GO Analysis of the Significant DEGs

To further clarify the potential pathway enrichment and functional annotation associated with the DEGs, GO analysis was performed with the clusterProfiler package (Yu et al. 2012). In the GO enrichment analysis, we sorted the genes according to the q value to select the top 10 genes.

#### 2.3.6 Single Sample Gene Set Enrichment

A single‐sample GSEA was performed using the clusterProfiler package, which enriched the top five up‐ and down‐regulated enrichment terms in the DEGs of NAFLD as well as the hub genes.

#### 2.3.7 Single‐Cell Sequencing Analysis

The results of reduction and clustering of GSE202379 were obtained using the results provided by Gribben C et al. A visual depiction of the gene expression patterns was generated through the use of violin plots, whereas uniform manifold approximation and projection (uMAP) plots were generated using Seurat functions, including VlnPlot and FeaturePlot (Stuart et al. 2019).

#### 2.3.8 Clinical Cross‐Sectional Study Analysis

In analyzing the baseline data, we utilized the Mann–Whitney U test for continuous variables (expressed as median and interquartile range [IQR]) and the chi‐square test for categorical variables (expressed as frequency and percentage [%]). In addition, we explored the association between cheese and fruit juice intake and NAFLD risk using different univariate and multivariate logistic regression model analyses. Model 1 was not adjusted for any variables, and model 2 was adjusted for sex, age, triglycerides (TG), low‐density lipoprotein cholesterol (LDL‐C) and high‐density lipoprotein cholesterol (HDL‐C) based on model 1. Model 3 further adjusted for aspartate aminotransferase (AST), blood urea nitrogen (BUN), gamma‐glutamyltransferase (GGT) and income from model 2 (Roark et al. 2022). To further explore the differences in the role of juice intake, cheese intake and NAFLD risk in different populations, we performed subgroup analyses by body mass index (BMI, < 25 or ≥ 25 kg/m^2^) and race (Mexican American, Hispanic, Non‐Hispanic White, Non‐Hispanic Black and others) groupings for subgroup analysis. All the results of this study were expressed using odd ratio (OR) and 95% confidence interval (CI). All analyses were performed in the R language environment (version 4.3.3), and two‐sided *p* values less than 0.05 were considered statistically significant.

## 3 Result

### 3.1 TSMR Analysis

**FIGURE 2:**
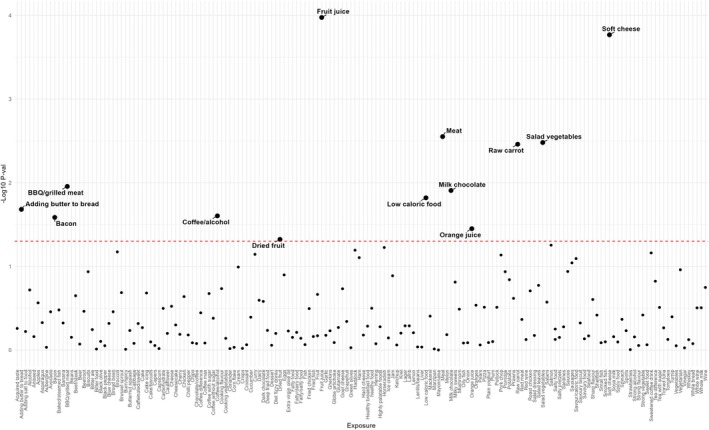
Distribution of *p*‐values for the causal relationship between 187 dietary preferences and NAFLD from the two‐sample Mendelian randomization analysis. The dashed line represents the critical value for the suggestive significance level, set at *p* = 0.05.

**FIGURE 3:**
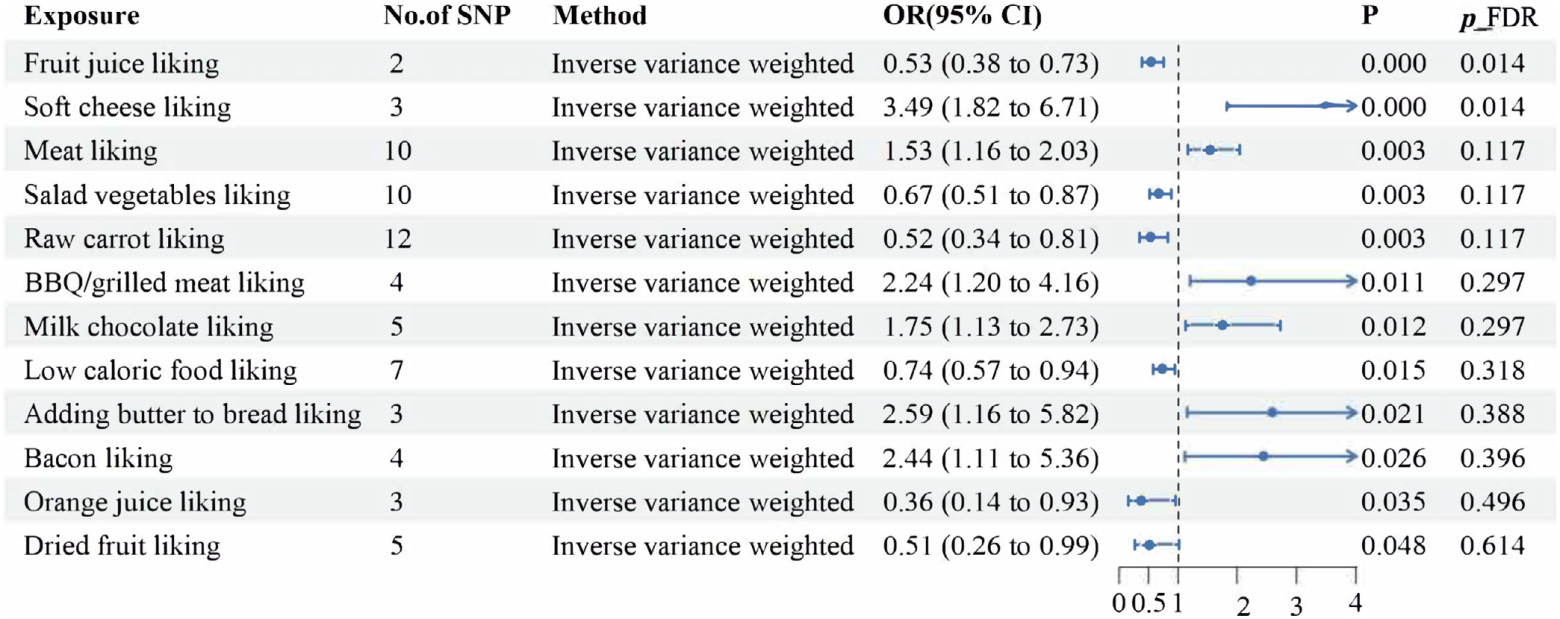
Forest plot of significant causal effects of dietary preferences on NAFLD risk as assessed by the inverse variance weighted approach. CI = confidence interval, FDR = false discovery rate, OR = odds ratio, SNP = single nucleotide polymorphism.

To explore the causal relationship between blood lipids and diet and the risk of NAFLD, we used 187 dietary preferences as exposure phenotypes and NAFLD as the outcome object, and performed TSMR analysis one by one, and all IVs included in the analysis are detailed in Table S1. In the results of TSMR, we used *p* = 0.05 as the threshold, and the results of *p* < 0.05 were regarded as potential positive results. Finally, we found that there was a potential causal relationship between 12 dietary preferences and NAFLD, and these 12 food preferences may lead to changes in the risk of NAFLD (Figures 2, 3). Among them, people who like fruit juice, vegetable salad, carrots, low‐calorie foods, orange juice, and dried fruits tend to have a lower risk of NAFLD; on the contrary, people who like to eat soft cheese, meat, processed meat, milk chocolate, bread with butter, and bacon have an increased risk of NAFLD. Subsequently, to ensure the stability of the results, we conducted a series of sensitivity analyses on all potential significant results. Cochran's Q test did not find significant heterogeneity, and the results of the MR‐Egger intercept test, leave‐one‐out method, and funnel plot did not find abnormal SNPs and horizontal pleiotropy. This suggests that our results are robust, and the results of sensitivity analyses are shown in Table S2.

**FIGURE 4:**
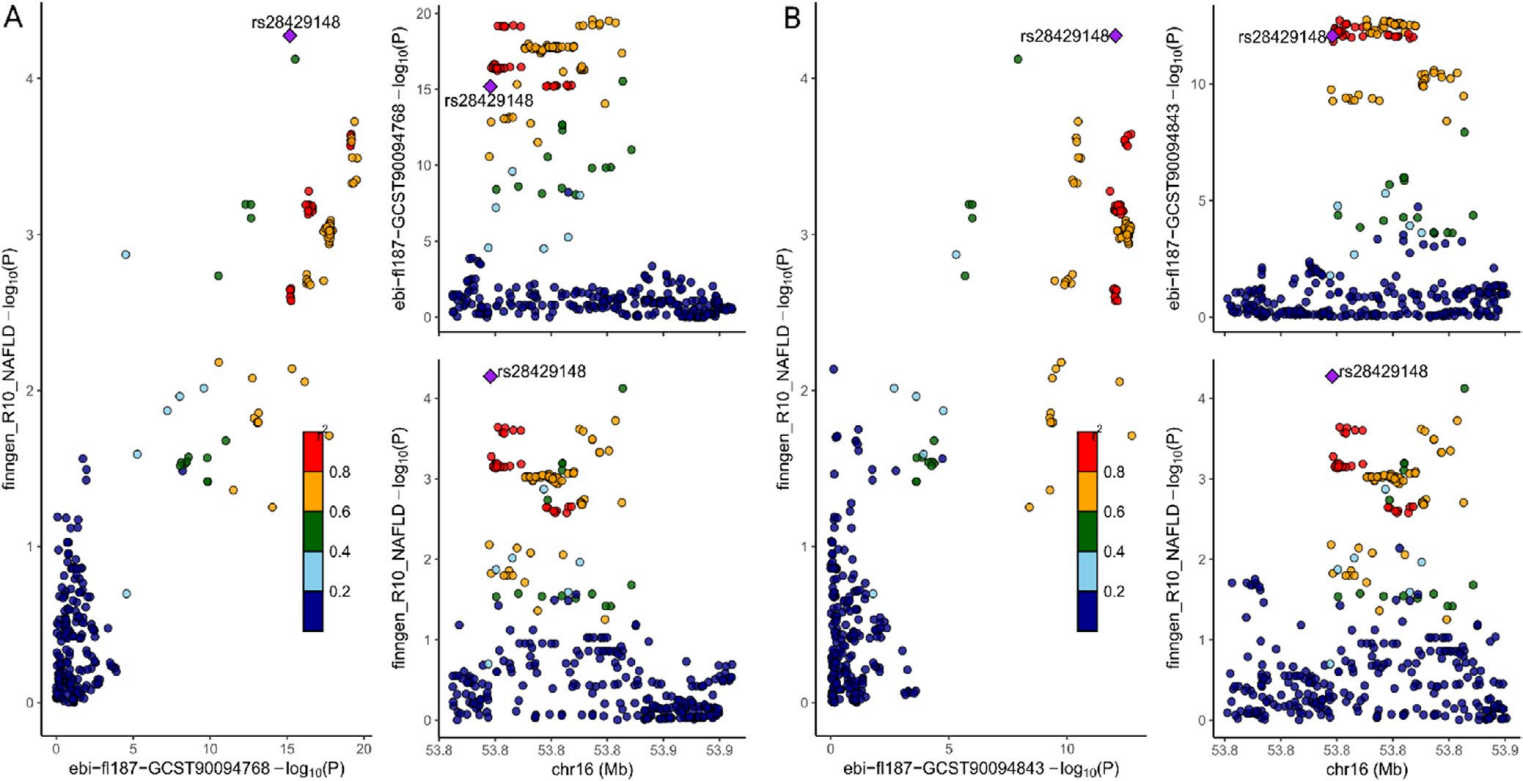
LocusCompare plots mark the loci driving causality in the results of co‐localization analyses of FDR‐corrected strong‐positive causality and distinguish false‐positive genes. (A, B) Distribution of SNPs—log10(p) in GWAS for soft cheese preference, juice preference, and NAFLD, respectively, is depicted, where rs28429148 is considered the mutant locus driving causality.

### 3.2 Two‐Step MR Analysis

On the basis of the TSMR results, we wanted to explore whether dietary preferences altered the risk of NAFLD by regulating the levels of inflammatory factors as in the current study. Therefore, we performed a two‐step MR randomization to explore the possible mediator proteins between the causal relationship between dietary preferences and NAFLD risk, using the 91 identified inflammatory proteins as potential mediators. Among the ten dietary preferences with significant causal relationships with NAFLD, we finally confirmed a mediating pathway. The specific mediation analysis results are (Table S1) shown in Table 1. Delta and Notch‐like epidermal growth factor‐related receptor (DNER) mediated 9%(95% CI = 3%–21%) of the causal effect of low‐calorie dietary preferences on the risk of NAFLD. Among them, low‐calorie dietary preferences has a negative causal effect on NAFLD (OR = 1.11, 95% CI = 1.00–1.23), while DNER has a negative causal effect on NAFLD (OR = 0.77, 95% CI = 0.62–0.95). This indicates that a low‐calorie diet reduces the risk of NAFLD by up‐regulating the level of inflammatory protein DNER, proving that dietary preferences change the risk of NAFLD by regulating the causal effect of inflammatory protein levels.

**TABLE 1:** The mediation proportion of inflammatory factor in the causal relationship between NFALD and diet.

Mediator	The effect of exposure on outcome OR (95% CI)	The effect of exposure on mediator OR (95% CI)	The effect of mediator on outcome OR (95% CI)	Mediated proportion
DNER	0.74 (0.58, 0.94)	1.11 (1.00, 1.23)	0.77 (0.62, 0.95)	0. 09 (0.03, 0.21)

### 3.3 Clinical Cross‐Sectional Data Analysis

To validate the strong positive MR result after multiple corrections for FDR that cheese preference increases NAFLD risk through genetic variation, whereas juice preference reduces NAFLD risk, we utilized clinical cross‐sectional data from NHANES to validate the association between the two intakes and NAFLD risk. First, from the baseline data of the NAFLD group and healthy controls, we found that age, CAP, ALT, AST, BUN, GGT, TC, TG, BMI, and LDL‐C were significantly higher in the NAFLD group than in the healthy control group, and there were more male patients, whereas the HDL‐C level was significantly lower than that in the healthy control group (Table S3). Most importantly, NAFLD patients consumed significantly less juice than healthy controls (*p* = 0.017).

In subsequent logistic regression analyses, we made incremental covariates adjustments in the 3 models to explore the relationship between cheese and fruit juice intake and the risk of NAFLD. In model 1, we did not adjust for covariates, and the results showed a significant negative association between only fruit juice intake and NAFLD risk (OR = 0.724, 95% CI = 0.583–0.9, *p* = 0.004). Subsequently, in multivariate logistic regression models for Models 2 and 3, we sequentially adjusted for sex, age, TG, LDL‐C, and HDL‐C; and sex, age, TG, LDL‐C, HDL‐C, AST, BUN, income and GGT. Results showed a similarly significant correlation between cheese intake and the risk of NAFLD (Model 2 [OR = 1. 306, 95% CI = 1.002–1.072, *p* = 0.039); Model 3 (OR = 1.039, 95% CI = 1.004, 1.075, *p* = 0.030]), and the negative correlation between juice intake and NAFLD risk remained significant. Subsequently, we conducted subgroup analyses by BMI and race in order to explore between‐group differences in different populations. In our results we found no significant differences in increased risk of NAFLD by cheese intake across BMI ranges, and in the subgroups of ethnicity there was only an increased risk of NAFLD by excessive cheese intake among Mexican Americans (OR = 1.161, 95% CI = 1.015, 1.327, *p* = 0.029) and Americans of other ancestry (OR = 1.115, 95% CI = 1.006, 1.235, *p* = 0.038) in whom excessive cheese intake led to an increased risk of NAFLD. Juice intake, on the other hand, was associated with an increased risk of NAFLD in BMI ≥ 25 kg/m^2^ (OR = 0.686, 95% CI = 0.515, 0.913, *p* = 0.010), Mexican American (OR = 0.422, 95% CI = 0.195, 0.915, *p* = 0.029) and Non‐Hispanic White (OR = 0.560, 95% CI = 0.366, 0.858, *p* = 0.008) demonstrated significant NAFLD protection (Table S5). This suggests that greater fruit juice intake in overweight and Mexican‐American and Non‐Hispanic White populations can significantly reduce NAFLD risk, and that Mexican‐Americans should also reduce cheese intake and thus NAFLD risk. In summary, higher juice intake and lower cheese intake were strongly associated with reduced risk of NAFLD in the United States population, which is consistent with the results of previous MR analyses (Table 2).

**TABLE 2:** Association between cheese intake, juice intake and risk of NAFLD.

Exposure	Outcome	Model 1			Model 2			Model 3		
		OR	95% CI	*p*	OR	95% CI	*p*	OR	95% CI	*p*
Cheese intake	NAFLD	1.013	[0.981, 1.046]	0.428	1.036	[1.002, 1.072]	0.039	1.039	[1.004, 1.075]	0.030
Juice intake	NAFLD	0.724	[0.583, 0.9]	0.004	0.654	[0.520, 0.823]	0.000	0.667	[0.529, 0.842]	0.001

### 3.4 Co‐Localization Analysis

We also performed FDR multiple corrections on all results to avoid false positive results interfering with our conclusions. Results with *p* < 0.05 after FDR correction were defined as strong positive results, representing a very significant causal relationship. Among the 12 potentially significant causal relationships, only juice preference (OR = 0.53, 95% CI 0.38–0.73, *p* = 0.000, *p*_FDR = 0.014) and soft cheese preference (OR = 3.49, 95% CI = 1.82–6.71, *p* = 0.000, *p*_FDR = 0.014) remained significant after correction, which proves that prefer juice has a significant negative causal effect on the risk of NAFLD, while preference soft cheese significantly increases the risk of NAFLD.

Colocalization analysis is a method to find gene loci that drive common causal relationships, and it can also test whether there are false positive causal relationships in MR results. There is evidence that colocalization analysis can help us better understand the role of gene loci in the association between diseases, and the colocalized targets found are excellent therapeutic targets, which can provide a strong basis for the development of targeted drugs and precision gene therapy. Therefore, in order to further explore the driving factors and potential therapeutic targets of the causal relationship between dietary preferences and NAFLD risk, we conducted colocalization analysis on the strong positive exposure cheese and juice preference and outcome NAFLD that were still significant after FDR correction, aiming to find specific causal driving SNPs. The results showed that the causal relationship between people with juice preference (PP.H3 = 0.045, PP.H4 = 0.852, PP.H3 + PP.H4 > 0.80) and soft cheese preference (PP.H3 = 0.085, PP.H4 = 0.728, PP.H3 + PP.H4 > 0.80) and NAFLD risk was driven by rs28429148, which corresponds to the fat mass, obesity‐related (FTO) gene (Figure 4 and Table S4). This indicates that the rs28429148 mutation of the FTO gene drives the negative causal relationship between juice preference and NAFLD risk and the positive causal relationship between soft cheese preference and NAFLD. Targeted regulation of the FTO gene containing this mutation site can effectively reduce the risk of NAFLD in people with FTO gene mutation and is expected to become a valuable new therapeutic target.

### 3.5 Enrichment Analysis and Single‐Cell Validation

**FIGURE 5:**
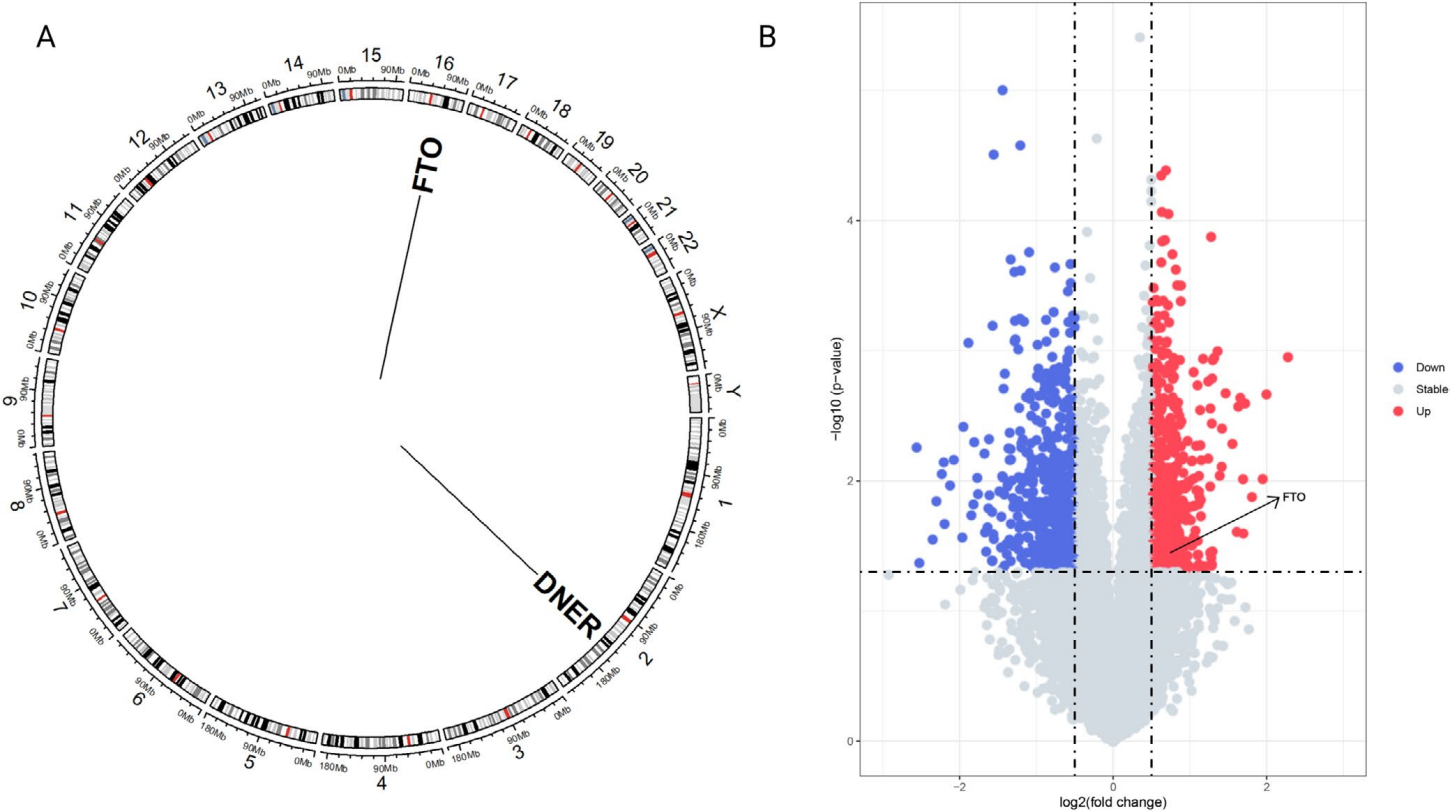
(A) Demonstrates the specific locations where the FTO and DNER genes, respectively, are present on human chromosomes. (B) Shows the expression content and expression trend of FTO and DNER genes in the human transcriptome dataset GSE260666.

**FIGURE 6:**
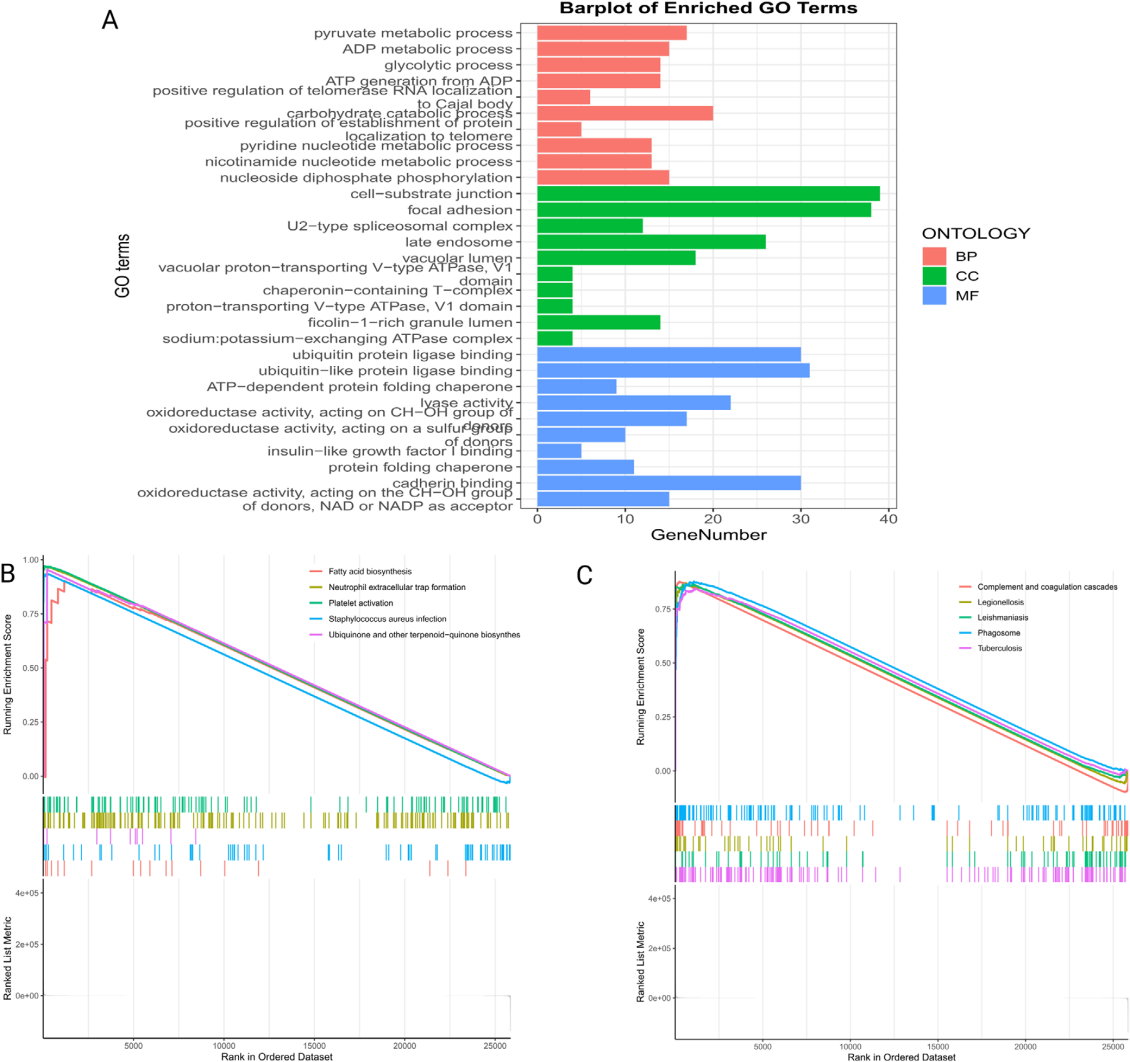
Enrichment analysis of the specific mechanism of action of FTO genes in NAFLD. (A) The results of GO enrichment analysis of the FTO gene. (B) Expression up‐regulation pathway results of GSEA enrichment analysis of the FTO gene. (C) Graph of pathway results of down‐regulated expression in GSEA enrichment analysis of FTO genes.

To further explore the specific mechanisms of the co‐localized variant target gene FTO and the mediator protein DNER‐regulated genes in NAFLD, we performed multiple bioinformatics analyses of DNER and FTO using the NAFLD sample RNA sequencing dataset GSE260666 and the single‐cell sequencing dataset GSE202379. The FTO gene is located on the long arm (q‐arm) of human chromosome 16, and its variation affects fat distribution and body weight changes in humans; DNER is located at 2q36.3 and is associated with the Notch pathway, which is involved in the development of nerve cells. The specific gene location is shown in Figure 5A. First, we screened the significantly over‐expressed and under‐expressed DEG genes in GSE260666, among which FTO was found to be significantly over‐expressed in NAFLD. However, DNER genes were not significantly over‐ or under‐expressed in transcriptome analysis, the results were displayed in the form of a volcano map (Figure 5B). Second, single gene enrichment analysis was performed on FTO to explore the mechanism of action of the FTO gene in NAFLD. The results of GO enrichment analysis showed that FTO mainly played an important role in pyruvate metabolism, ADP metabolism, glycolysis, and carbohydrate catabolism through ATP‐dependent proteins, oxidoreductases, and lyase activities (Figure 6A). The results of GSEA enrichment analysis showed that the FTO gene was mainly involved in up‐regulating fatty acid biosynthesis and neutrophil‐extracellular capture network formation in NAFLD population, and down‐regulating glycosaminoglycan and coagulation cascade reactions (Figure 6B).

**FIGURE 7:**
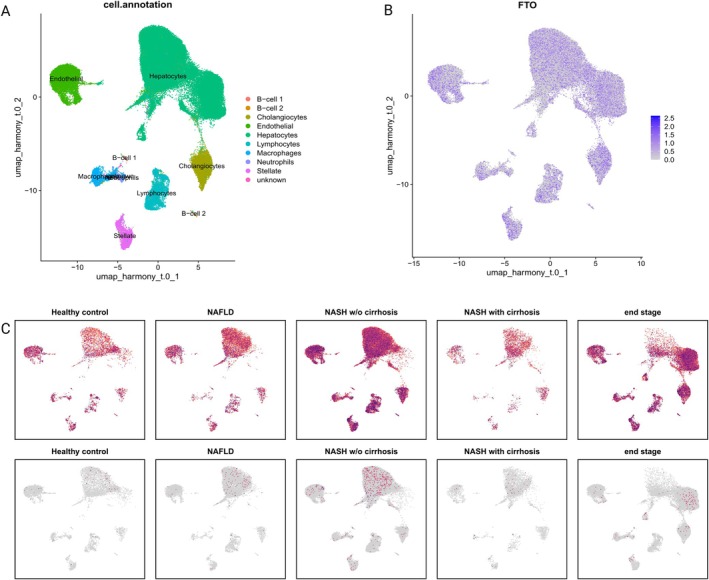
Expression and distribution of FTO and DNER genes in the NAFLD single‐cell dataset GSE202379. (A) Cellular subpopulation maps of tissues after downscaling in GSE202379. (B) Expression and distribution plots of FTO in NAFLD patient tissues in different cellular subpopulations. (C) Expression and distribution plots of FTO and DNER genes in cellular subpopulations in tissues from healthy controls and NAFLD samples at different stages of the disease.

Finally, we verified the expression and distribution of FTO and DNER in tissues in the single‐cell dataset GSE202379 of NAFLD patient tissues. We used the Seurat package to analyze the dataset, and the uMAP graph was used for dimensionality reduction clustering. The results of cell clustering are shown in Figure 7A. NAFLD tissues were divided into 10 cell subpopulations, namely B‐cell 1, B‐cell 2, Cholangiocytes, Endothelial, Hepatocytes, Lymphocytes, Macrophages, Neutrophils, Stellate, and unknown cells. FTO was distributed in all cell populations and was highly expressed in NAFLD tissues at different pathological stages, especially in NASH with cirrhosis and in the end‐stage state (Figure 7B,C), which again demonstrated that the FTO gene plays an important role in the pathogenesis of NAFLD. In contrast, the DNER gene, despite higher expression in the disease state than in healthy controls, remained at a low overall expression level, with higher expression only in hepatocytes under NASH in cirrhosis and in the end state (Tables [Link], [Link]).

## 4 Discussion

This study is the first to comprehensively investigate the causal relationships and possible intermediate mediators between dietary preferences and NAFLD risk in a population via multi‐omics analysis and clinical cross‐sectional analysis. First, TSMR analyzed 187 dietary preferences and NAFLD revealed that populations with preferences for fruit juices, vegetable salads, carrots, low‐calorie foods, oranges, and dried fruits had potentially lower NAFLD risk. Preferences for soft cheeses, meats, processed meats, milk chocolate, buttered breads, and bacon could have a potentially positive causal effect on NAFLD risk. Second, we performed a two‐step MR analysis of potential positive causal effects, and the results revealed that the inflammatory protein DNER mediated the causal effect of a low‐calorie diet on reducing NAFLD risk. Third, after FDR correction of the results, we found a strong positive causal relationship between people who preferred soft cheese and fruit juice and NAFLD. The results were similarly confirmed in a cross‐sectional study of the NHANES data, where preference for cheese increased the risk of NAFLD, and vice versa, preference for fruit juice decreased it. Fourth, colocalization analyses for the 2 strong positive causal relationships revealed that the variant locus rs28429148 in the FTO gene also drives the positive and negative causal effects of cheese preference and juice preference on NAFLD risk, respectively. Finally, we validated the expression of the FTO gene in the NAFLD population and explored the specific mechanism of action via RNA transcriptome enrichment analysis and single‐cell analysis.

To comprehensively explore the causal relationship between dietary preferences and NAFLD risk, we included a total of 187 dietary preferences from GWAS data as exposures and analyzed NAFLD as an outcome via the TSMR, which revealed 12 significant potential causal relationships. Among the results, we observed that meat, processed meat, bacon, milk chocolate, buttered bread and soft cheese preferences may genetically causally increase the risk of NAFLD. Among these, meat, processed meat and bacon have been shown to be significantly associated with an increased risk of NAFLD in existing studies. Meat, especially red meat, is a major component of the Western dietary pattern, and although red meat is rich in protein, vitamins, iron, and other micronutrients, it contains extremely high levels of cholesterol, heme iron, and saturated fat that may directly contribute to the onset and progression of NAFLD, which is in line with the findings of our study (Ivancovsky‐Wajcman et al. 2022; Hashemian et al. 2021). Multiple studies have shown that red meat intake contributes to NAFLD, insulin resistance, and metabolic syndrome and induces a range of oxidative stress responses, with obesity mediating the relationship between red meat and increased risk of NAFLD (Wang et al. 2023; Zelber‐Sagi et al. 2018). In addition, red meat may also lead to intestinal flora disorders and subsequent disruption of hepatic lipid metabolism and carbohydrate metabolism, and the intestinal metabolites of L‐carnitine in red meat, trimethylamine and trimethylamine‐N‐oxide, directly contribute to the development of atherosclerosis and NAFLD. The high heat and dry cooking required for processed meats and bacon results in the conversion of myoglobin in red meat to glycosylation end products (AGEs), an increase in which has been shown to be a causative agent of hepatic stellate cell activation. This also correlates positively with insulin resistance and NASH risk (Zelber‐Sagi et al. 2018). Similarly, processed meat products often contain excessive levels of preservatives and sodium components (nitrites or nitrates), all of which are strongly associated with NAFLD. In another study, NASH patients were given dark chocolate and milk chocolate, and significant improvements in oxidative stress, endothelial function and insulin resistance were found in the dark chocolate group, whereas no such improvements were observed in the milk chocolate group (Malhi and Loomba 2016). This suggests that dark chocolate consumption may have a potential benefit on NAFLD risk, whereas milk chocolate has no significant benefit or harm, which is inconsistent with our results. The difference in the benefits of dark and milk chocolate on NAFLD may be able to be explained by the lipid‐lowering, antioxidant and anti‐apoptotic properties contained in cocoa, the main component of dark chocolate (Magrone et al. 2017; Munteanu and Schwartz 2023). The associations between the consumption of different categories of chocolate and NAFLD risk need to be further explored in the future. Our results also revealed a similarly increased risk of NAFLD in people who preferred bread spread with butter, possibly because butter is rich in high levels of saturated fat of animal origin, which exacerbates insulin resistance (Rivellese et al. 2002). In addition, butter can increase arachidonic acid levels in the liver and thus mediate chronic inflammation in the liver (Hussein et al. 2007). Instead, people with a high risk of NAFLD may consider consuming high‐quality fats such as peanut oil and olive oil, which have been shown in studies to have excellent antioxidant and insulin resistance‐improving effects on the body, ultimately reducing the risk of NAFLD (Munteanu and Schwartz 2023).

Among the protective dietary preferences, we found that people who preferred vegetable salads and carrots had a lower risk of NAFLD, which is in line with the current mainstream opinion. Experts believe that the beneficial effect of the Mediterranean diet on NAFLD patients is due to the high vegetable content of the diet, which has been shown in several studies to protect against NAFLD risk factors such as obesity and type 2 diabetes (Quetglas‐Llabrés et al. 2023). An analysis of different vegetables revealed that vegetables rich in components such as beta‐carotene and flavonoids, such as carrots, tomatoes, pumpkins, and spinach, are beneficial for metabolic abnormalities such as NAFLD, type 2 diabetes, and other diseases. These vegetables possess a greater ability to down‐regulate adipogenic genes, lowering the risk of NAFLD in the form of fat accumulation in the liver as a result of dyslipidemia (Yilmaz et al. 2015). In addition, β‐carotene reduces the risk of NAFLD through its powerful ability to inhibit the inflammatory response, reduce antioxidant inactivity, and significantly improve insulin resistance, which is consistent with our findings (Balbuena et al. 2022). According to the TSMR results, orange juice preference also had a negative causal effect on NAFLD risk, possibly because of its raw material, citrus. Researchers believe that citrus and its derivatives have the potential to become important functional foods, as they contain a wide range of active substances that are beneficial to human health. Among them, pectin‐rich citrus pulp and polyphenol‐rich citrus peel significantly reduced triglycerides, LDL, and total cholesterol levels in mice and reduced adipocyte volume in mice fed a high‐fat diet in animal studies (Hu et al. 2021).

It also has a strong anti‐inflammatory effect, which is achieved by inhibiting inflammation‐related enzymes such as Cyclooxygenase‐2 (COX‐2), which ultimately reduces the production of NAFLD‐inducing inflammatory factors such as Tumor Necrosis Factor‐Alpha (TNF‐α) and Interleukin‐6 (IL‐6). In addition, citrus essential oil is rich in limonene and polyphenol extracts, which has antimicrobial and antioxidant properties. It can also improve the accumulation of liver fat under a high‐fat diet by regulating the intestinal flora, ultimately inhibiting the occurrence and progression of NAFLD (Wang et al. 2022). Finally, there are few studies of dried fruit diets to reduce the risk of NAFLD. Aesculus chinensis Bunge ripe dried fruits have been found to protect the liver from oxidative damage and reduce lipid accumulation through the active substance hesperidin, but its efficacy needs to be further investigated in patients with or at risk for NAFLD (Yu et al. 2023).

We performed a two‐step MR analysis across 12 causal associations in which dietary preferences exhibited a potentially positive association with NAFLD. The results revealed that low‐calorie dietary preferences could reduce the risk of NAFLD by increasing DNER expression. Low‐calorie dietary patterns such as low‐fat and ketogenic habits have been demonstrated to significantly reduce body weight and the levels of various inflammatory factors in humans (Zhou et al. 2021). This may be due to weight and dietary modifications that improve the body's oxidative stress, flora function and insulin resistance (Ruiz‐Otero and Kuruvilla 2023). In an animal study of obese mice, a calorie‐restricted, low‐fat diet resulted in a significant decrease in the expression of a number of chemokines and inflammatory cells, such as IL‐1Ra, IL‐2, IL‐6, Monocyte Chemoattractant Protein‐1 and C‐X‐C Motif Chemokine Ligand 16 (Pardo et al. 2021). Jonasson et al. and Castaldo et al., on the other hand, reported that a ketogenic diet was more effective than a low‐fat diet in reducing the levels of inflammatory factors, such as IL‐6 and TNF‐α, in patients with NAFLD who were overweight for 6 months (Jonasson et al. 2014; Castaldo et al. 2021). Systemic inflammation is an important etiologic factor in the development of several chronic diseases such as NAFLD, type 2 diabetes, and Alzheimer's disease, and low‐calorie diets have been suggested to be effective in reducing the risk of NAFLD because of their anti‐inflammatory effects, which is consistent with our findings. On the basis of existing studies, we analyzed and found that the inflammatory protein DNER may mediate the negative causal relationship between low‐calorie dietary preferences and the risk of NAFLD, a novel mediator. DNER is an inflammation‐associated membrane protein expressed mainly in the cerebellum, pituitary gland, and adrenal glands; it has been shown to be a potential therapeutic target for gastric cancer and osteoarthritis in previous studies. The correlation between waist circumference and body mass index with distal sensorimotor polyneuropathy has not been investigated in patients with NAFLD (Costa et al. 2024; Malouf et al. 2014; Schlesinger et al. 2019). Recently, researchers have reported that DNER is also distributed in human adipose‐derived mesenchymal stem cells (hAMSCs) and that down‐regulation of DNER expression leads to accelerated lipid accumulation in hAMSCs. This is because inhibition of DNER upregulates C/EBPd expression and keeps hAMSCs in a quiescent state, which bypasses the MCE and in turn produces more fat and larger lipid droplets, ultimately resulting in abnormal accumulation and obesity (Ponce‐de‐Leon et al. 2022; Park et al. 2010). The accelerating effect of down‐regulated DNER on adipogenesis ultimately leads to a greatly increased risk of NAFLD, and conversely, promoting DNER protein expression may reduce the risk of NAFLD. In addition, DNER has been suggested by researchers to be associated with abnormal glucose metabolism, insulin resistance, and hepatic gluconeogenesis. DNER knockout mice showed reduced insulin secretion and impaired insulin signaling in animal experiments. This is possibly because DNER gene deficiency predisposes them to defects in the pancreatic islet β‐cell intermuscular actin dynamics as well as increased hepatic gluconeogenesis (Ruiz‐Otero and Kuruvilla 2023; Li et al. 2024). Similarly, increased DNER expression may reduce insulin resistance and diabetes risk, thus indirectly reducing the risk of NAFLD onset and progression. In the validation of the transcriptome and single‐cell analysis results, we did not observe significantly high expressions of the DNER gene in the transcriptome. However, in the single‐cell analysis, we found that expression of the DNER gene was greater in NAFLD tissues than in healthy control tissues, which was consistent with the results of the MR analysis. The high expression of DNER in the pathological tissues of NAFLD patients may be a result of negative feedback in the disease state that up‐regulates DNER expression and promotes its protective effect, but this mechanism remains unspecified. In summary, the DNER protein is expected to be a new therapeutic target for NAFLD; nevertheless, no study has explored the specific role of DNER in the pathogenesis of NAFLD. Whether low‐calorie dietary preferences can up‐regulate DNER expression needs to be further explored.

After applying FDR multiple corrections to the TSMR results, the causal associations between cheese preference and fruit juice preference and NAFLD risk remained significant, which we regarded as strong positive results. We then validated the MR results with a clinical cross‐sectional study using the NHANES database, which revealed that cheese intake was significantly positively associated with NAFLD risk, whereas fruit juice intake was significantly negatively associated with NAFLD risk, which was consistent with the results of the MR analysis. Existing studies have suggested differences in the associations between different dairy products and NAFLD risk, with cheese consumption increasing NAFLD risk and milk consumption reducing NAFLD risk (Yuzbashian et al. 2023). Cheese potentially increases the risk of NAFLD due to the large amount of fat components rich in saturated fatty acids and cholesterol. The excessive intake of these components worsens insulin resistance, systemic inflammation, and abnormalities in lipid metabolism within the body, ultimately leading to an increased risk of NAFLD (Wu et al. 2024). There is still controversy about the ability of fruit juices to reduce the risk of NAFLD, with juices such as pomegranate and bergamot demonstrating a significant protective effect against NAFLD, and the opposite is true for some other fruits (Naomi et al. 2023; Musolino et al. 2020). This may be due to the varying fructose contents of different fruits. Excessive intake of fruit juices and fructose can counteract the beneficial effects of vitamins, flavonoids, and fiber on NAFLD, thus increasing the risk of NAFLD (Lee et al. 2022; Buziau et al. 2022). Through colocalization analyses, we subsequently identified the specific gene locus rs28429148, which is located on the FTO gene, drives the causal association between cheese and juice preferences and an increased risk of NAFLD. The FTO proteins belong to the family of iron‐ and 2‐oxoglutarate‐dependent dioxygenases, which are biologically active through demethylation and play a role mainly in the regulation of metabolism (Martin Carli et al. 2018). The FTO gene has been found to play a key role in the development of NAFLD in several existing studies, and in a GWAS, study the FTO gene had a positive causal effect on obesity and blood glucose levels, which is in line with the major pathways of action of FTO in NAFLD revealed in our transcriptome enrichment analysis (Chen, Du, et al. 2023; Mizuno 2018). In clinical studies, the expression of the FTO gene and protein in NAFLD patients was significantly greater than that in healthy controls. Additionally, in animal experiments, the upregulation of the FTO gene in mice significantly increased food intake, whereas FTO‐deficient mice presented significant decreases in adiposity and body weight. The main mechanism of hepatic fat accumulation caused by FTO may involve the over‐expression of Peroxisome Proliferator‐Activated Receptor Alpha, a regulator of fatty acid oxidation, by FTO, which reduces lipid metabolism and promotes lipogenesis in hepatocytes through the FTO/SREBP1c/CIDEC pathway, ultimately leading to NAFLD (Chen et al. 2018; Wei et al. 2022). Currently, researchers have identified four FTO allele genetic variants (rs1421085, rs8050136, rs3751812, and rs9939609) that may increase the risk of NAFLD. The present study revealed that rs28429148 is a novel FTO variant capable of influencing the risk of NAFLD in terms of colocalization MR analysis. This study reveals that rs28429148 is a novel FTO variant that can affect NAFLD risk from the perspective of colocalization MR analysis and provides a reliable explanation for the drivers of the significant causal relationship between cheese and juice preferences and NAFLD risk (Rivera‐Iñiguez et al. 2023). In addition, the high expression profile of the FTO gene in NAFLD and the specific mechanism of action were validated via both transcriptome analysis and single‐cell analysis, which further enhances the reliability of the results. Our findings provide a novel perspective on lifestyle intervention approaches to inhibit NAFLD occurrence and progression through dietary control, providing strong evidence that FTO can significantly influence NAFLD risk. However, research exploring the specific mechanisms underlying the causal relationship between cheese and fruit juice and FTO expression is still lacking and needs to be explored in depth in the future.

There are several advantages of this study. First, we used dietary GWASs containing 187 dietary preferences, which is the most extensive coverage of dietary data at present. The latest GWAS data with the largest sample size were also selected for NAFLD, which avoids the small sample size limitation of traditional clinical studies and ensures the reliability of the results. Second, we used multiomics research methodology, which combines a variety of MR analyses, clinical cross‐sectional studies, and bioinformatics analysis, to demonstrate the causal relationship between dietary preferences and NAFLD risk and specific intermediate mediators from multiple dimensions, which increases the reliability of the results and provides potential targets for drug therapy. Third, we provide novel evidence for genetic causal aspects of the relationship between diet and NAFLD and conduct a series of sensitivity analyses to avoid confounding by confounders and reverse causation. However, limitations of this study also exist. First, limited by the single ethnic source of the GWAS dataset, our results can only be applied to European ancestry populations at present, and more GWAS data from other ancestry sources will be needed in the future to validate the findings with MR analysis. Second, we did not observe significant expression of the DNER gene via transcriptome analysis. This does not mean that DNER is similarly low at the protein level, and future bioinformatics studies related to the validation of the expression and the role of DNER proteins in NAFLD populations are still needed. Finally, more basic experimental validation that we need in the future (e.g., knockdown/over‐expression of DNER in NAFLD cells or animal models) helps us to gain a deeper understanding of the specific mechanisms by which dietary preference affects NAFLD risk.

## 5 Conclusion

The present multi‐omics study revealed significant causal effects and possible intermediate mediators between multiple dietary preferences on NAFLD risk by combining MR, Bayesian co‐localization, clinical cross‐sectional studies, and bioinformatics analysis. Among these, DNER mediated the reducing effect of low‐calorie diets on NAFLD risk, whereas the variant locus rs28429148 of FTO drove the protective effect of fruit juices on NAFLD as well as the risky effect of soft cheeses on NAFLD. Our findings highlight the importance of personalized dietary intervention strategies in the prevention and management of NAFLD, reveal two potential therapeutic targets, FTO and DNER, and provide new directions for clinical treatment of NAFLD.

## Author Contributions

**Qingan Fu:** wrote the main manuscript text. **Jierui Liu:** acquired the data. **Zhekang Liu** and **Tianzhou Shen:** data analysis. **Qingyun Yu** and **Huangxin Zhu:** prepared figures. **Shisheng Wu** and **Rixiang Liu:** prepared tables. **Yanze Wu** and **Xiaoping Yin:** prepared supplementary material. **Deju Zhang, Xiao Liu, Jianping Liu, Jing Zhang** and **Peng Yu:** revise the manuscript. All authors have read and agreed to the published version of the manuscript.

## Conflicts of Interest

The authors declare no conflicts of interest.

## Supporting information


Figure S1.

Table S1.

Table S2.

Table S3.

Table S4.

Table S5.

## Data Availability

The datasets used in this study are all publicly available. The dietary preference‐related genome‐wide association study (GWAS) data were sourced from the UK Biobank, NAFLD‐related GWAS data were obtained from the FinnGen study, and inflammatory protein GWAS data were provided by the Olink laboratory across multiple cohorts. Additionally, transcriptomic data from GSE260666 and GSE202379 were retrieved from the GEO database, and clinical data were collected from the 2017–2020 National Health and Nutrition Examination Survey (NHANES). All data are publicly available, and ethical approvals were obtained in the original studies. The data used in this research can be accessed via the following links: https://www.ncbi.nlm.nih.gov/gds, https://www.finngen.fi/en/access_results, https://doi.org/10.1038/s41590‐023‐01588‐w, and https://doi.org/10.1038/s41467‐022‐30187‐w.
