# Supplementary material for: Multi‐Omics Analysis Reveals Causal Relationships and Potential Mediators Between Dietary Preferences and Risk of NAFLD
Source: Food Sci Nutr. 2025 Jun 23;13(6):e70446. doi: 10.1002/fsn3.70446 (PMC12183393; doi:10.1002/fsn3.70446)

**Supplementary Figures Legend**

**Figure S1** Causal relationship between juice liking and NAFLD

**Figure S2** Causal relationship between soft cheese liking and NAFLD

**Figure S3** Causal relationship between meat liking and NAFLD

**Figure S4** Causal relationship between salad vegetables liking and NAFLD

**Figure S5** Causal relationship between raw carrot liking and NAFLD

**Figure S6** Causal relationship between BBQ/grilled meat liking and NAFLD

**Figure S7** Causal relationship between milk chocolate liking and NAFLD

**Figure S8** Causal relationship between low caloric food liking and NAFLD

**Figure S9** Causal relationship between adding butter to bread liking and NAFLD

**Figure S10** Causal relationship between bacon liking and NAFLD

**Figure S11** Causal relationship between orange juice liking and NAFLD

**Figure S12** Causal relationship between dried fruit liking and NAFLD

**Figure S1** Causal relationship between juice liking and NAFLD


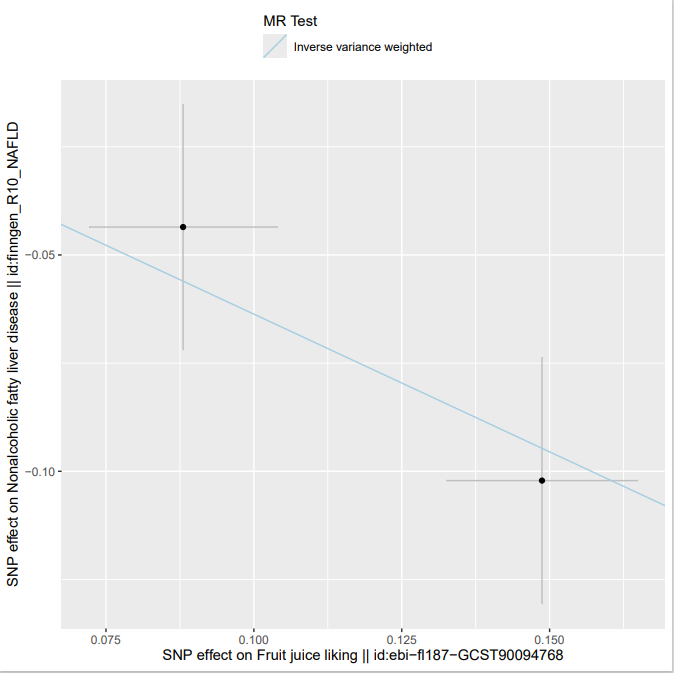

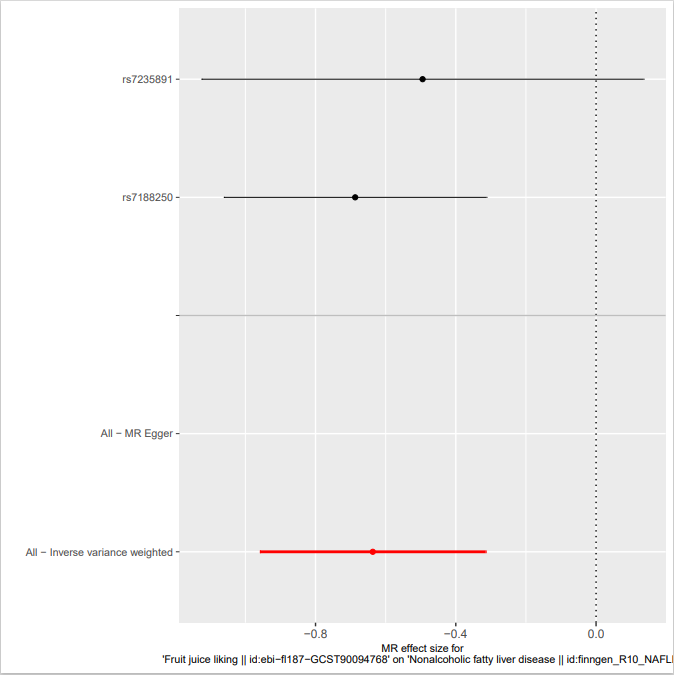


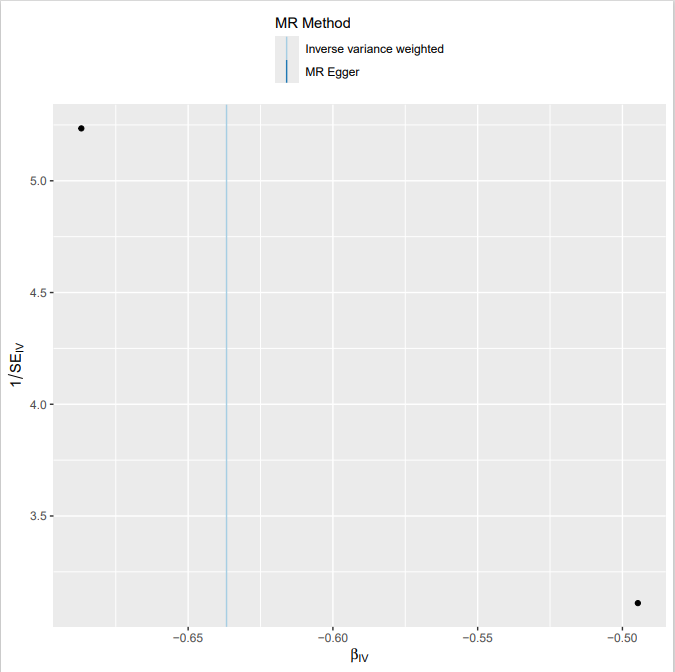


**Figure S2** Causal relationship between soft cheese liking and NAFLD


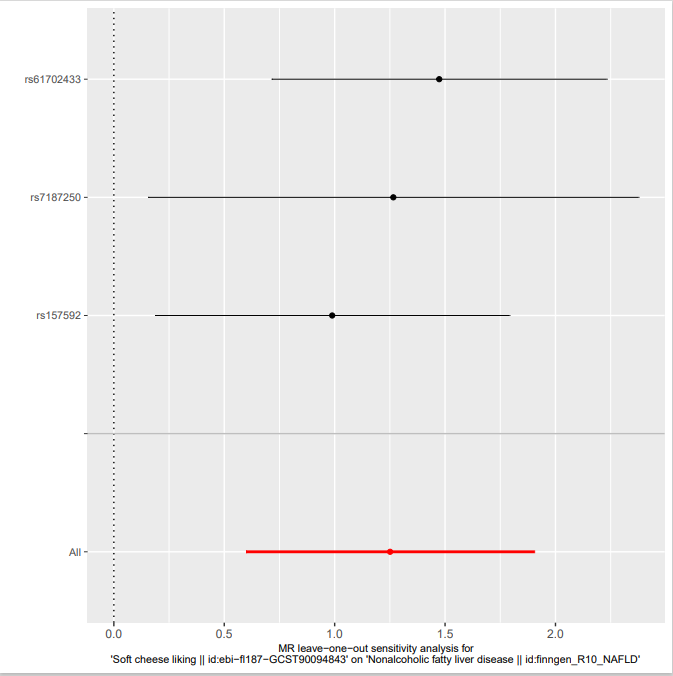

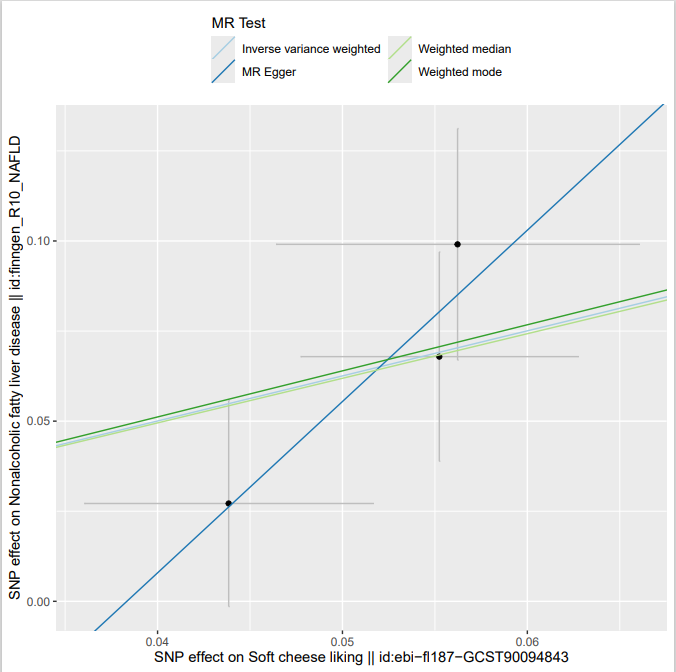


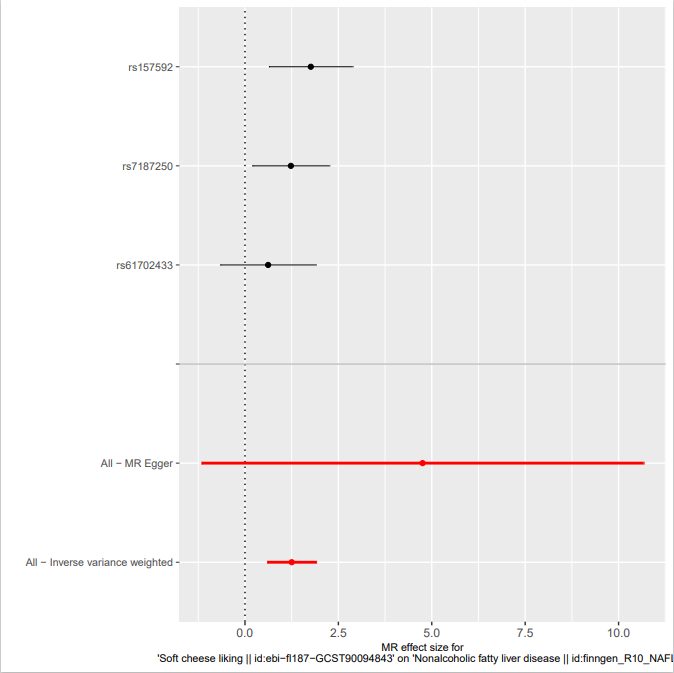

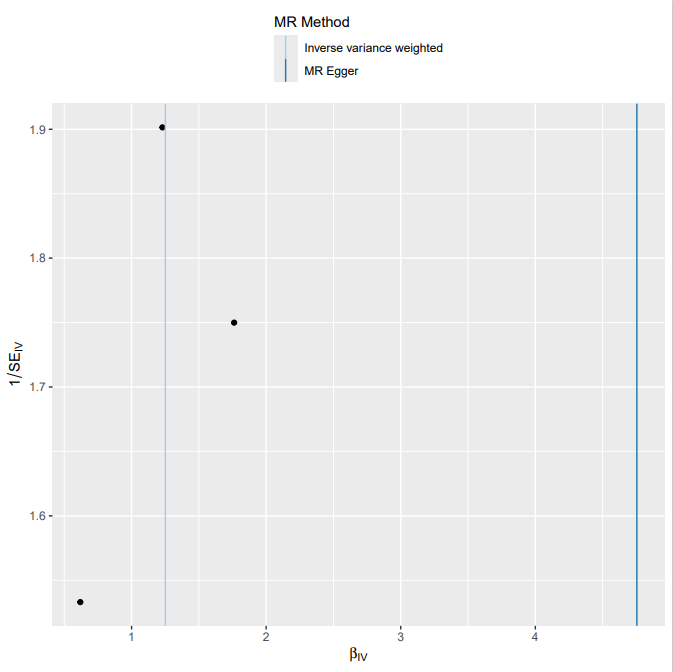


**Figure S3** Causal relationship between meat liking and NAFLD


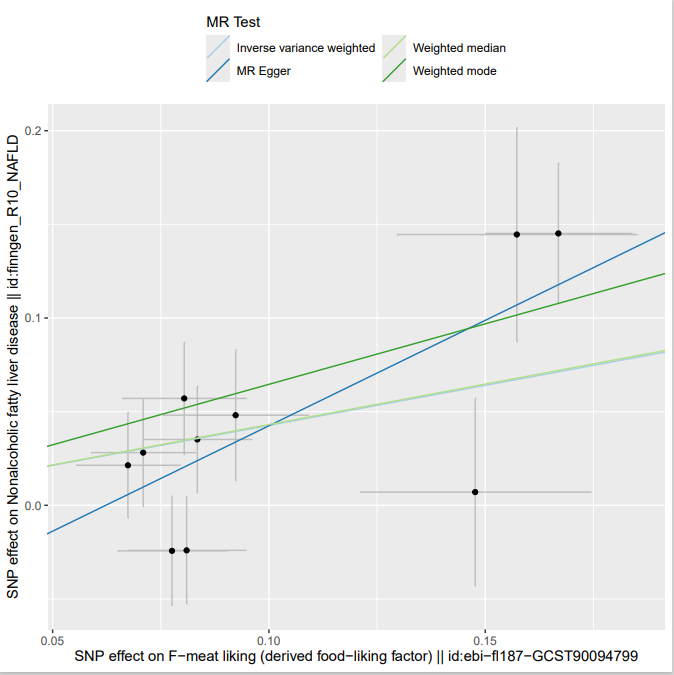

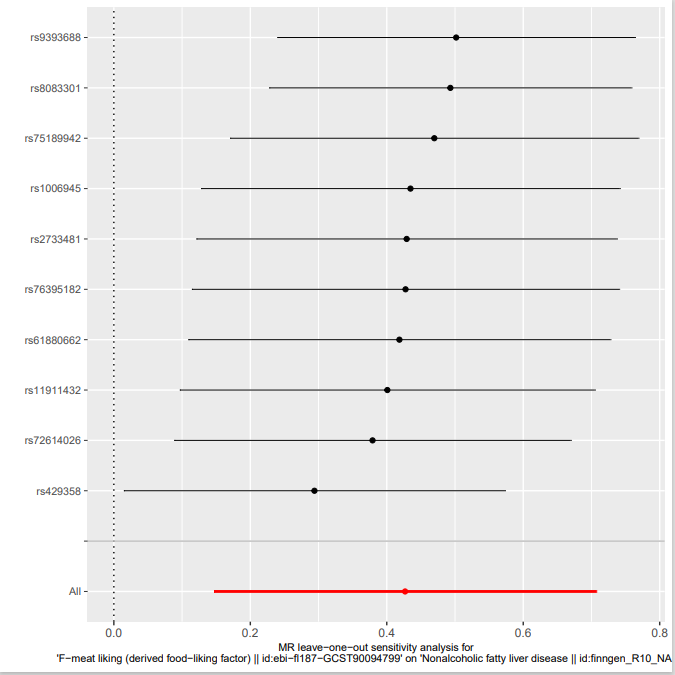


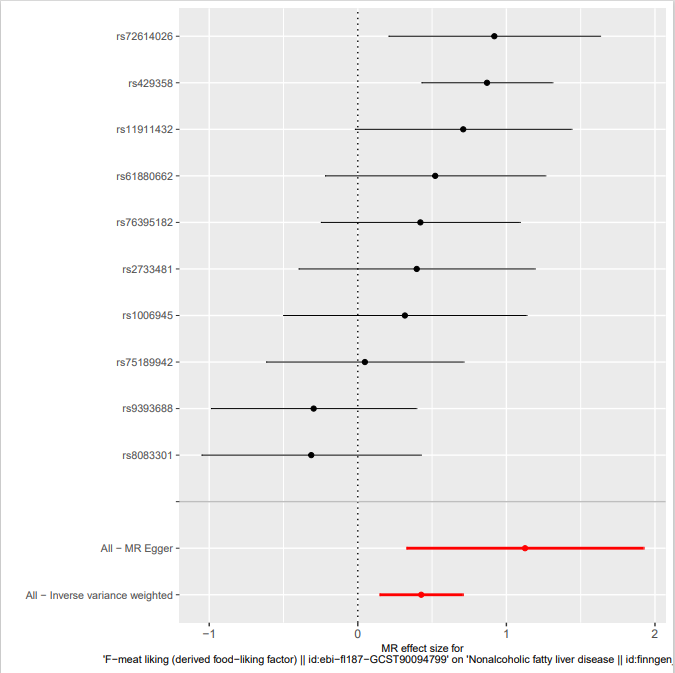

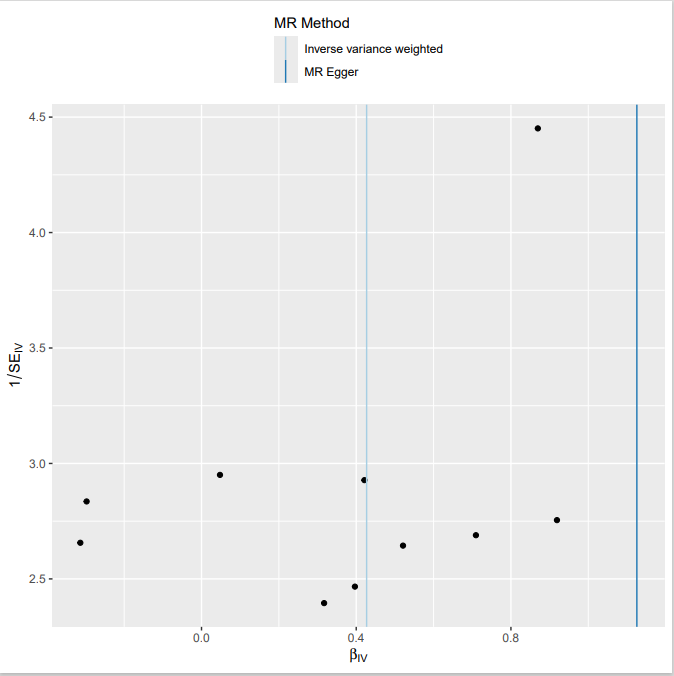


**Figure S4** Causal relationship between salad vegetables liking and NAFLD


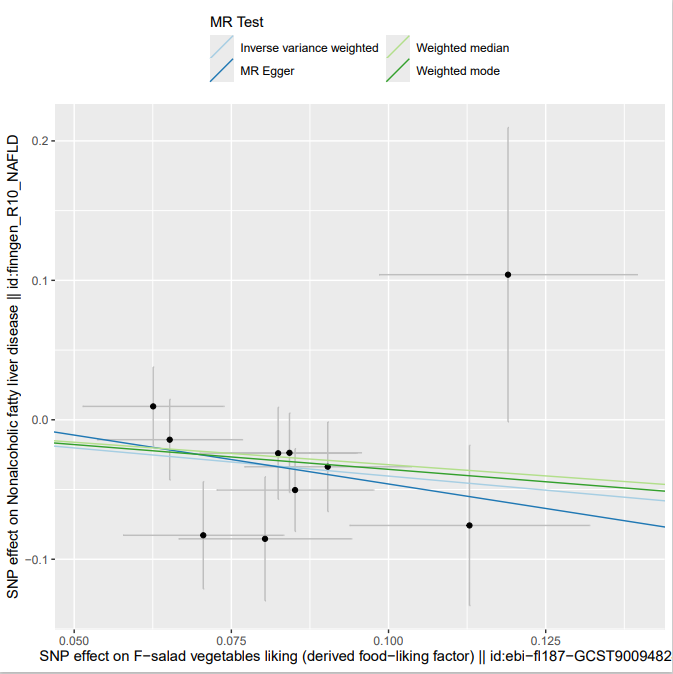

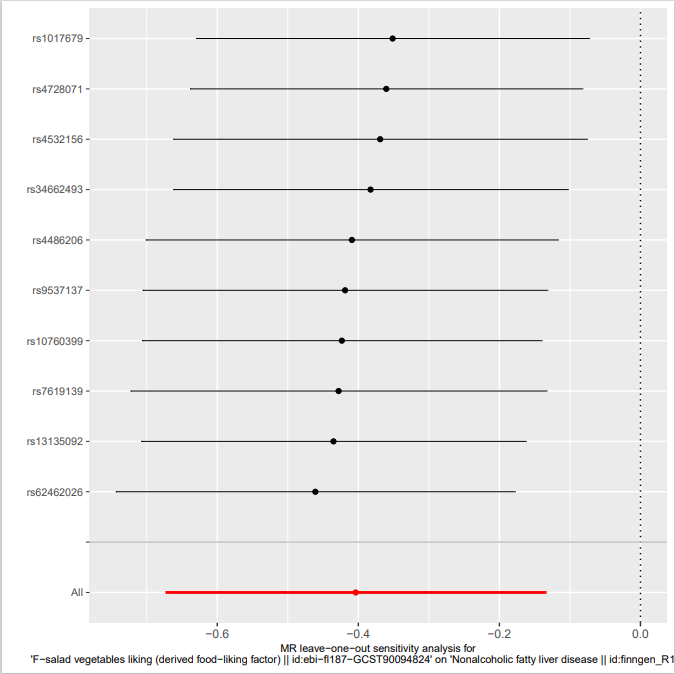


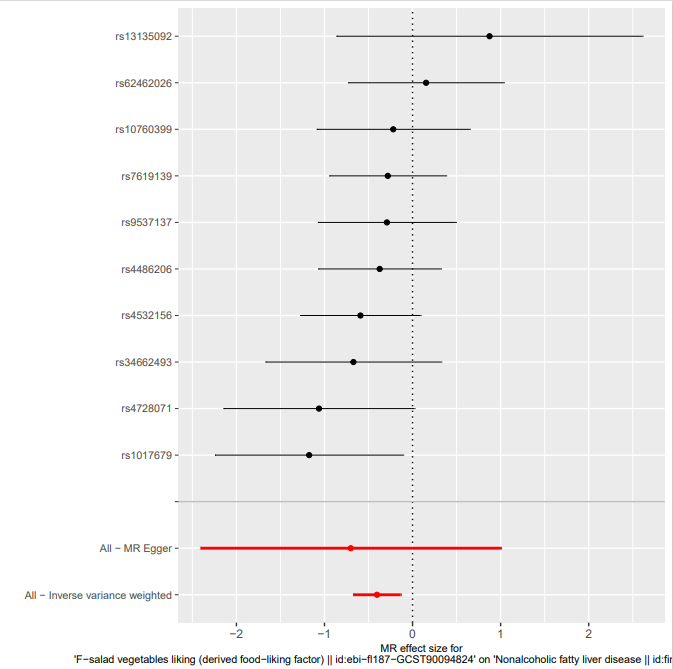

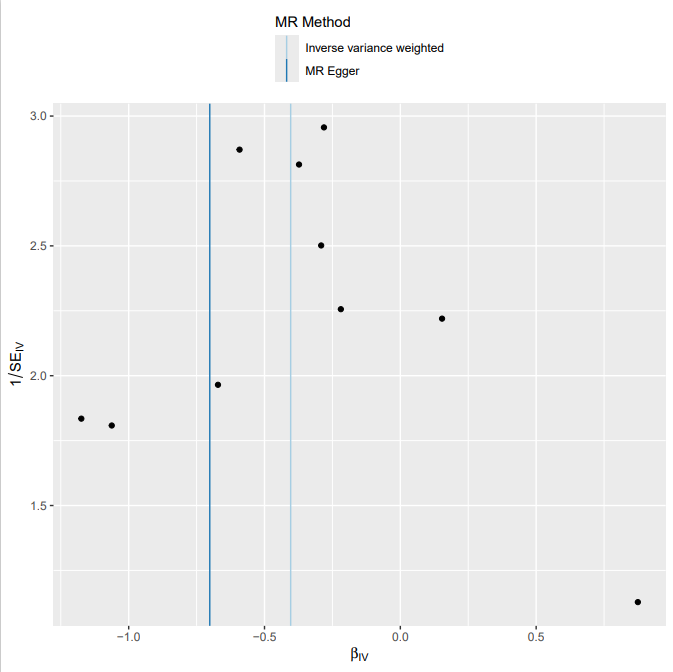


**Figure S5** Causal relationship between raw carrot liking and NAFLD


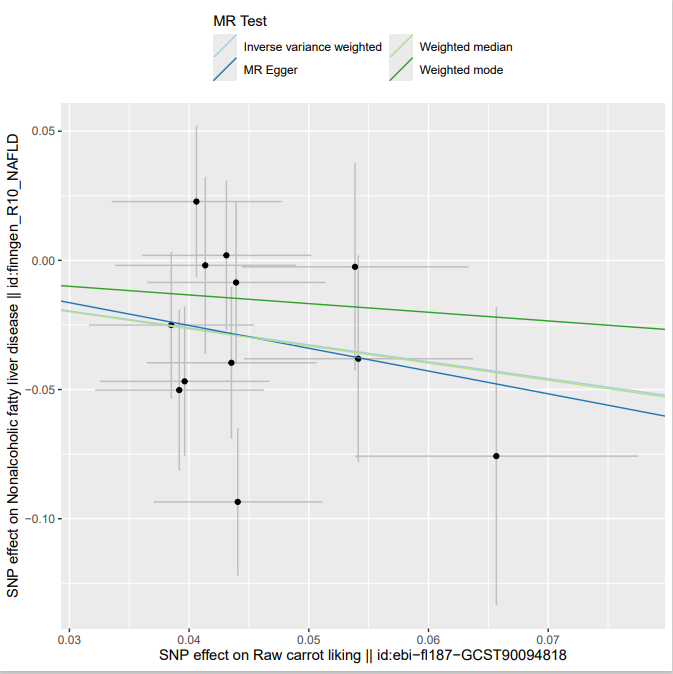

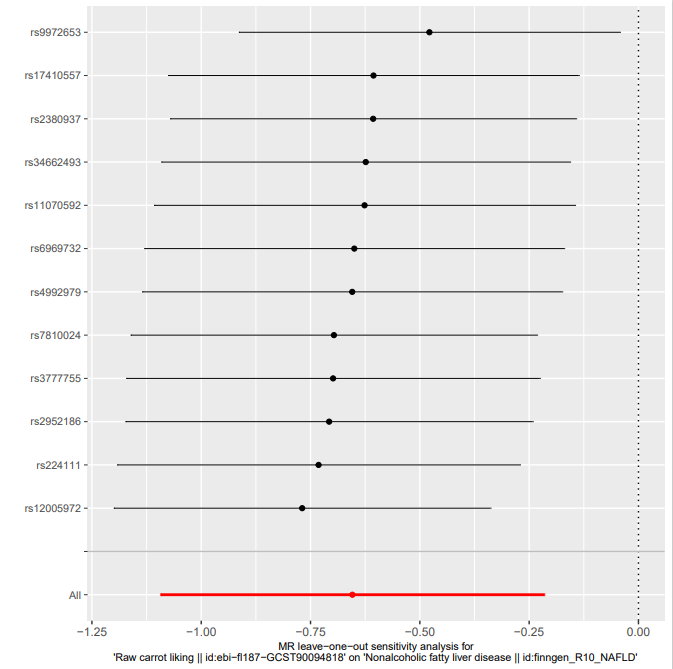


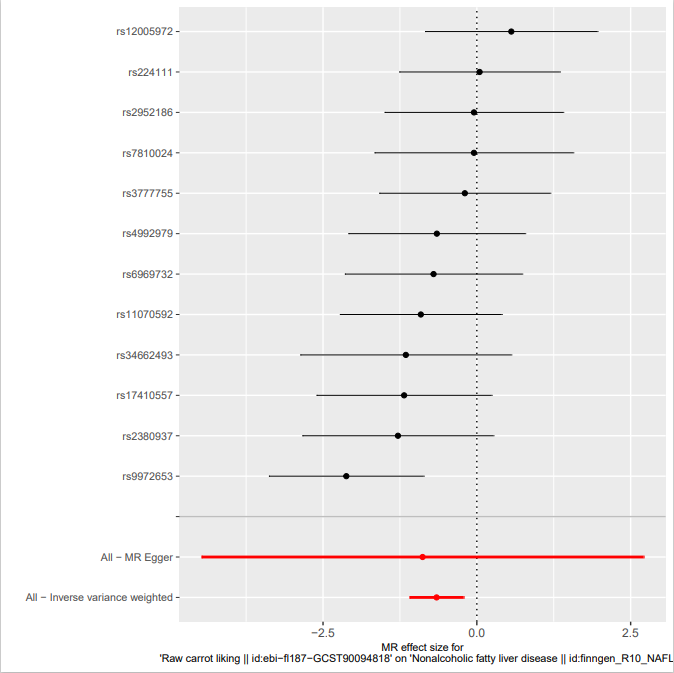

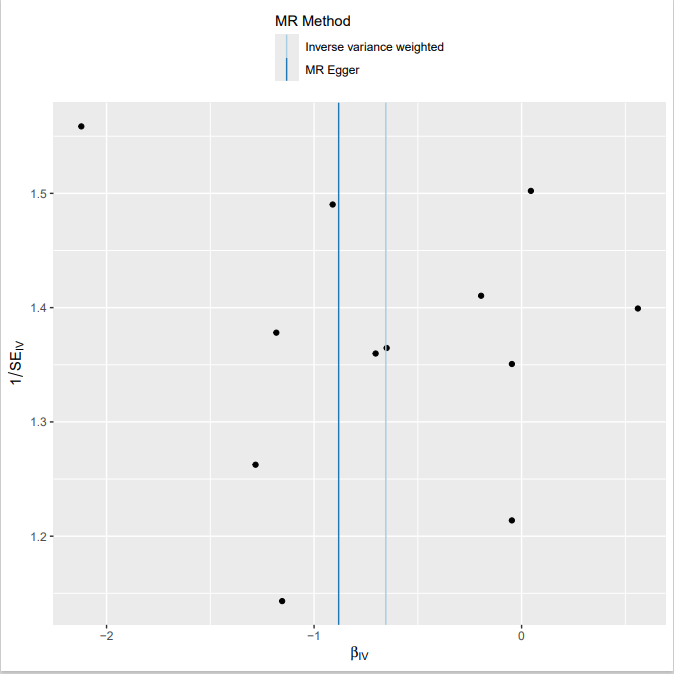


**Figure S6** Causal relationship between BBQ/grilled meat liking and NAFLD


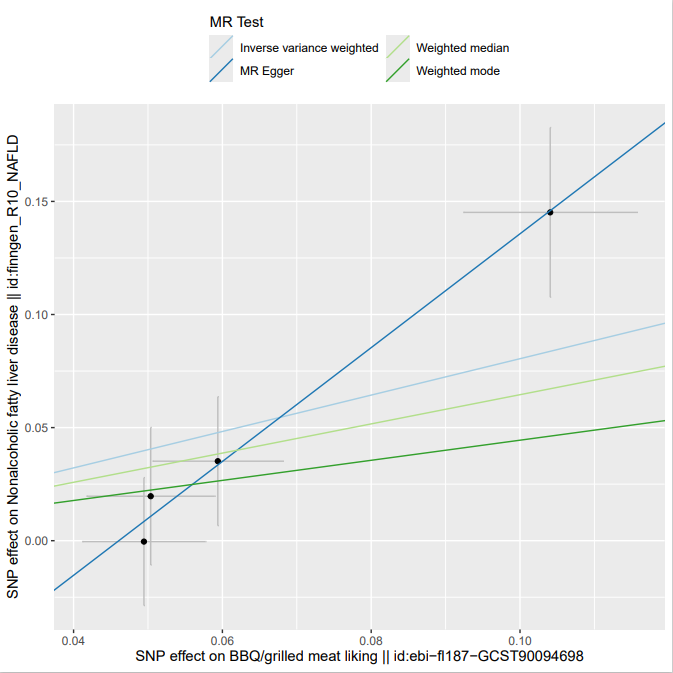

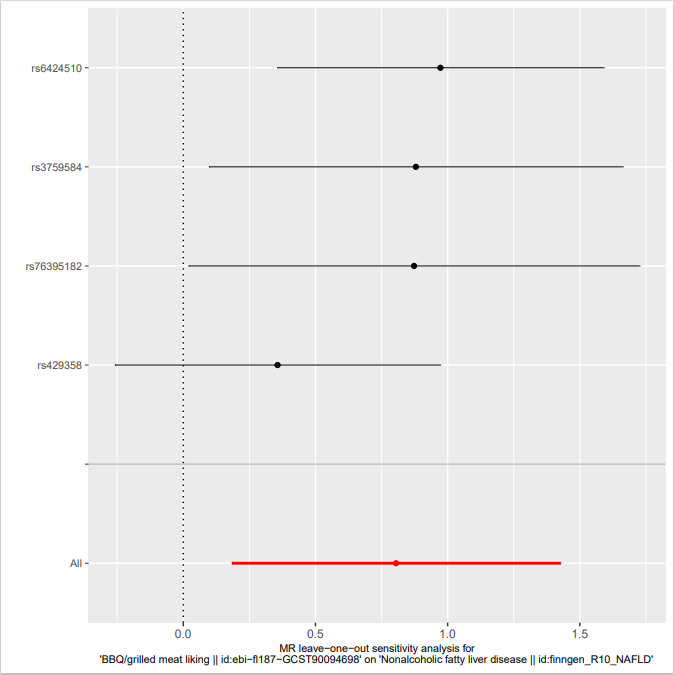


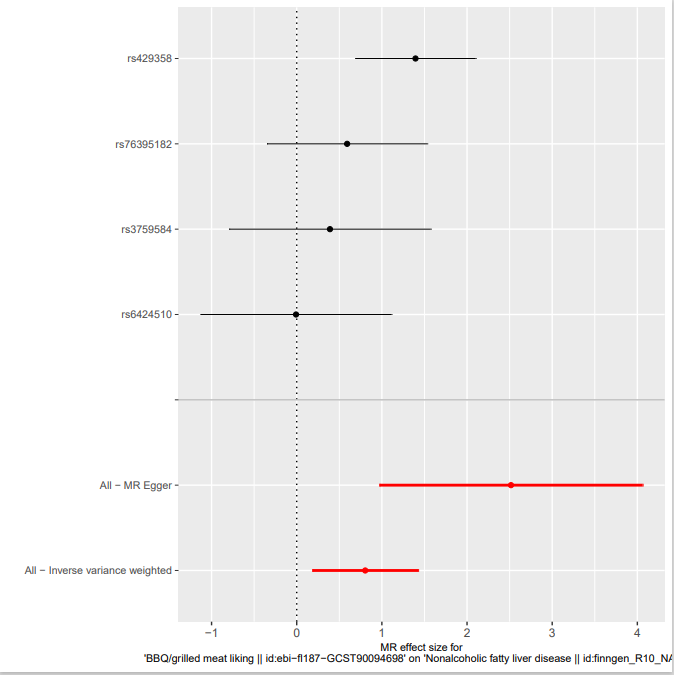

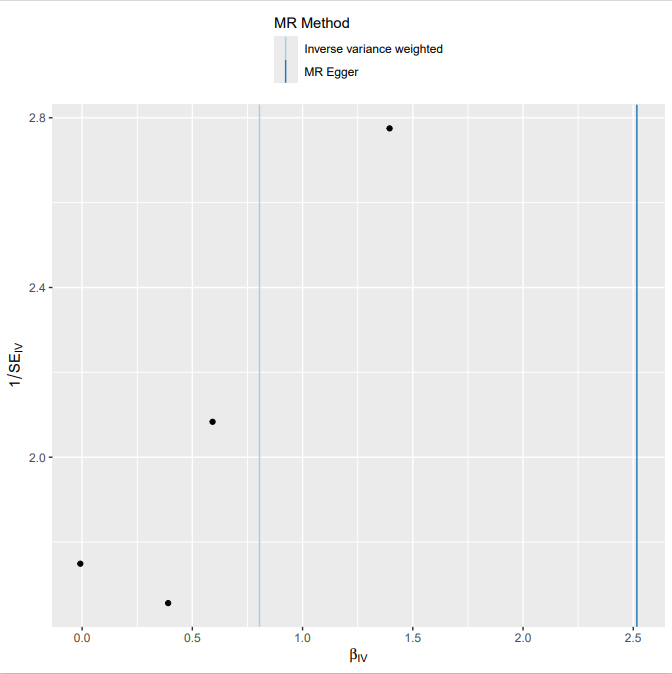


**Figure S7** Causal relationship between milk chocolate liking and NAFLD


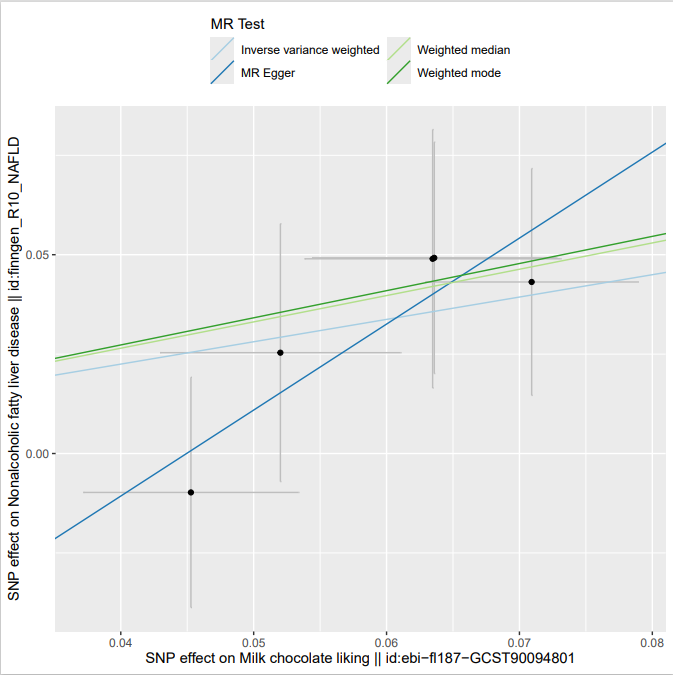

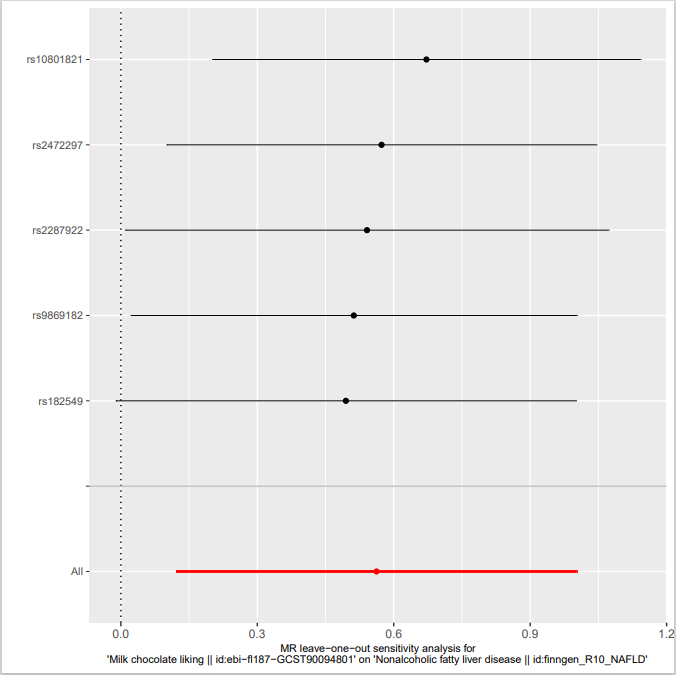


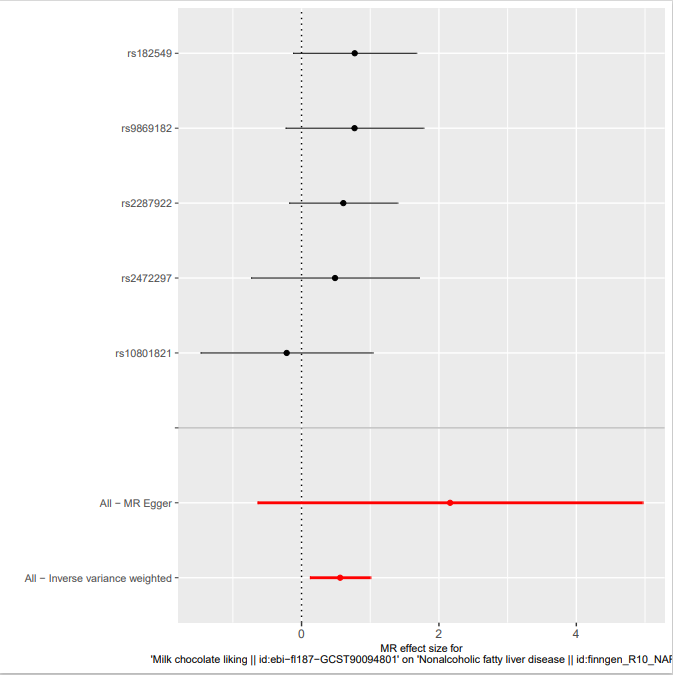

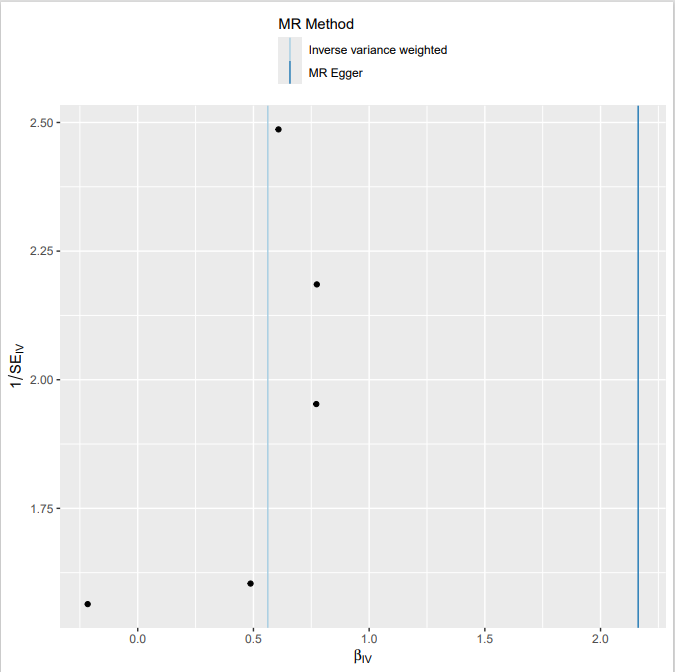


**Figure S8** Causal relationship between low caloric food liking and NAFLD


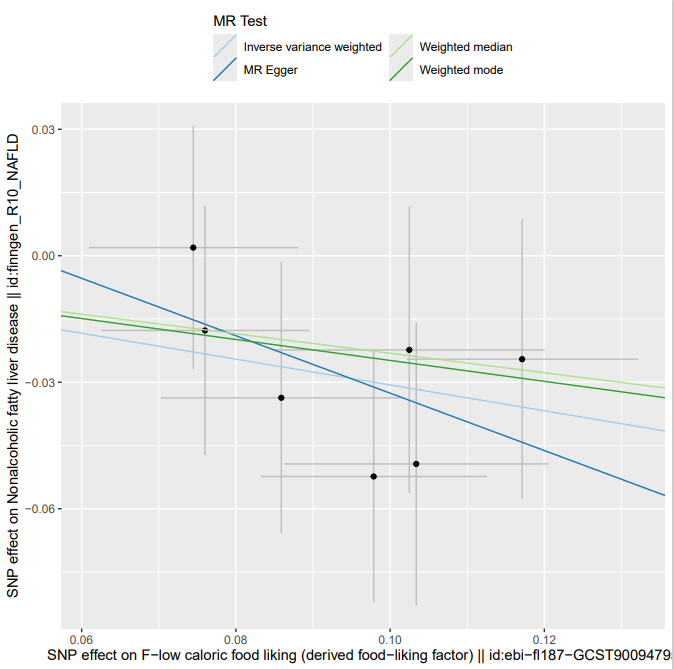

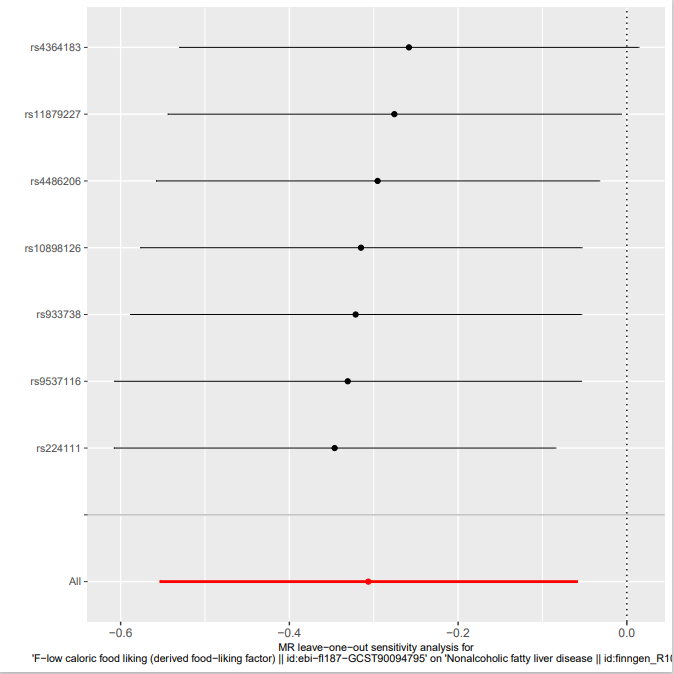


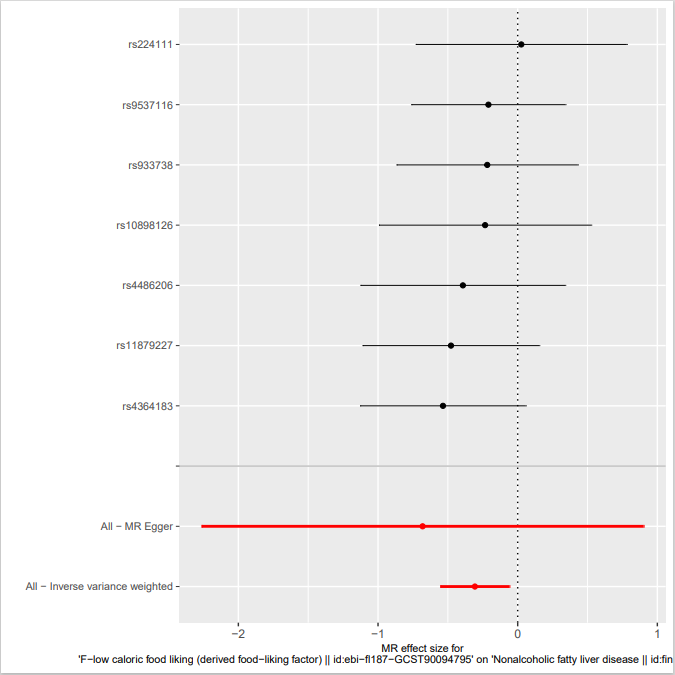

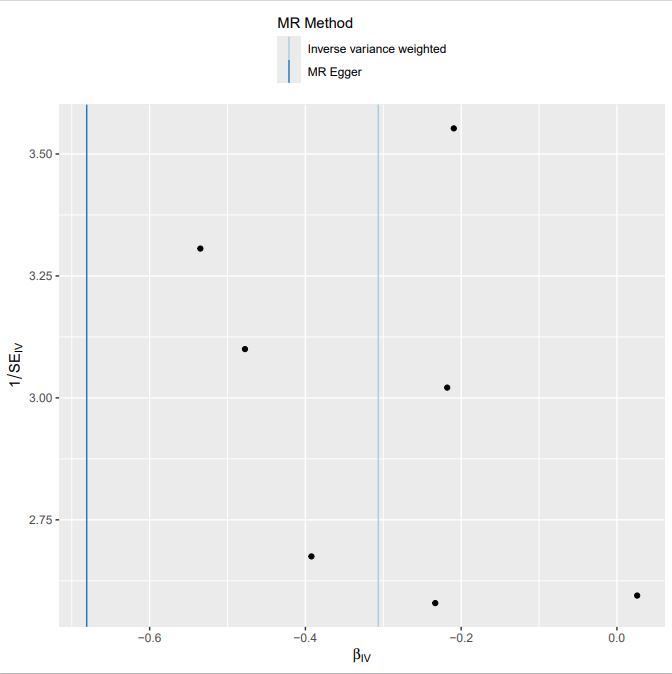


**Figure S9** Causal relationship between adding butter to bread liking and NAFLD


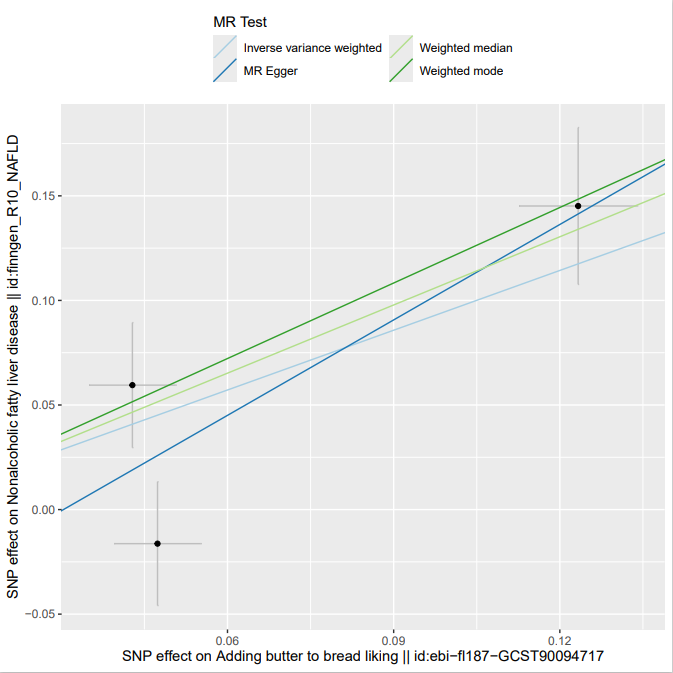

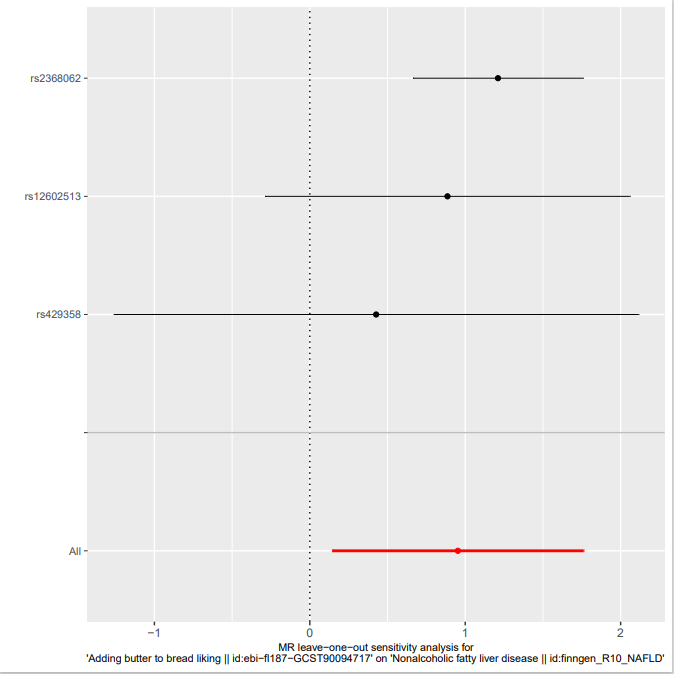


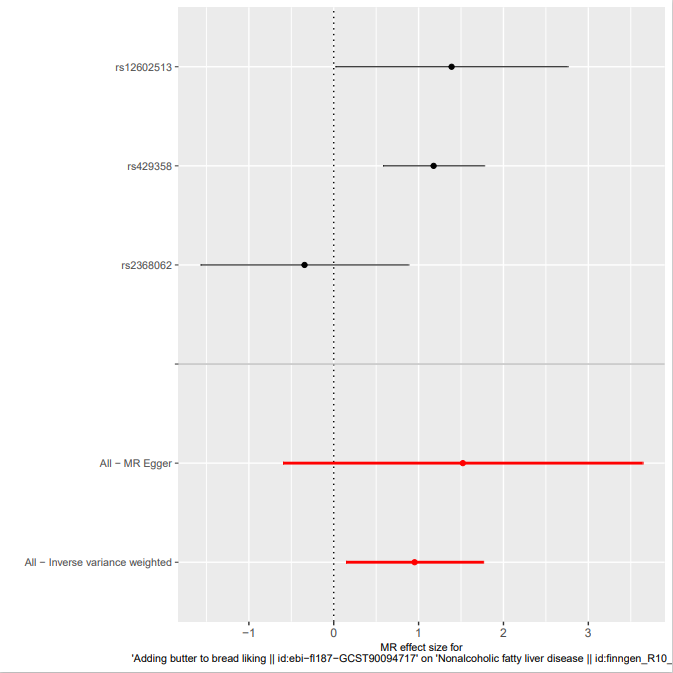

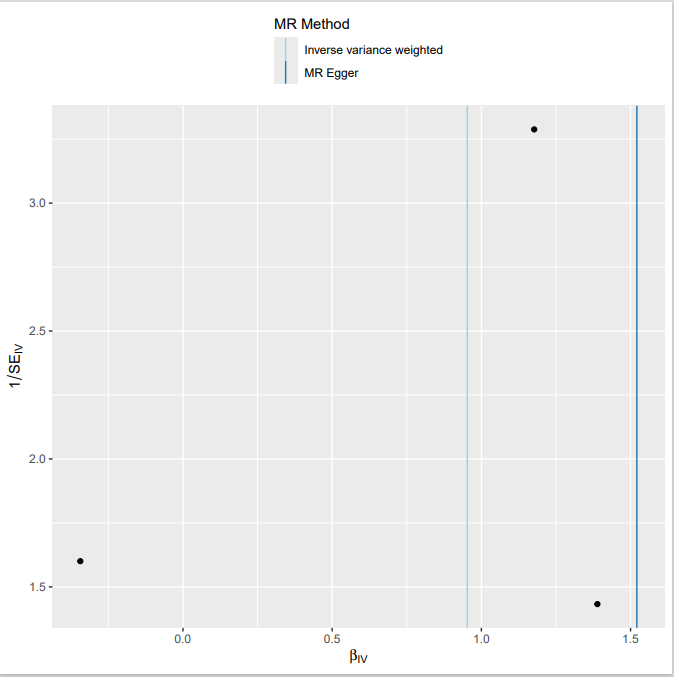


**Figure S10** Causal relationship between bacon liking and NAFLD


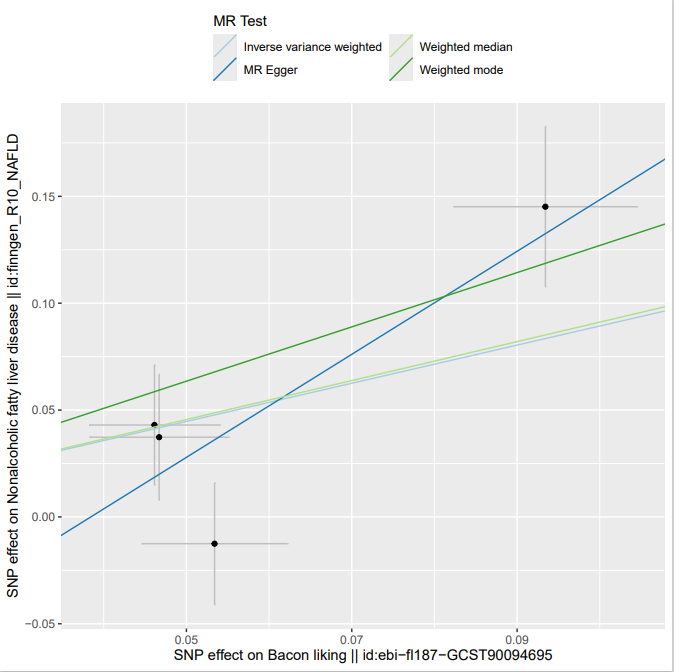

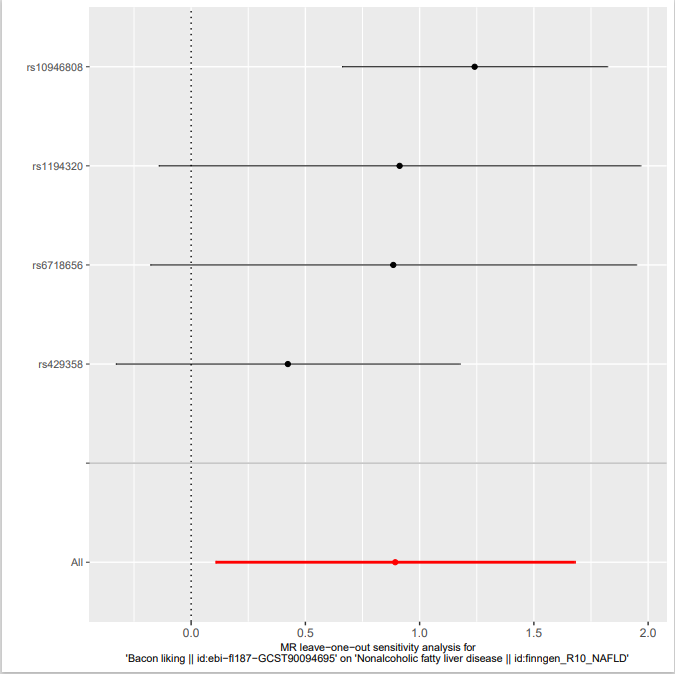


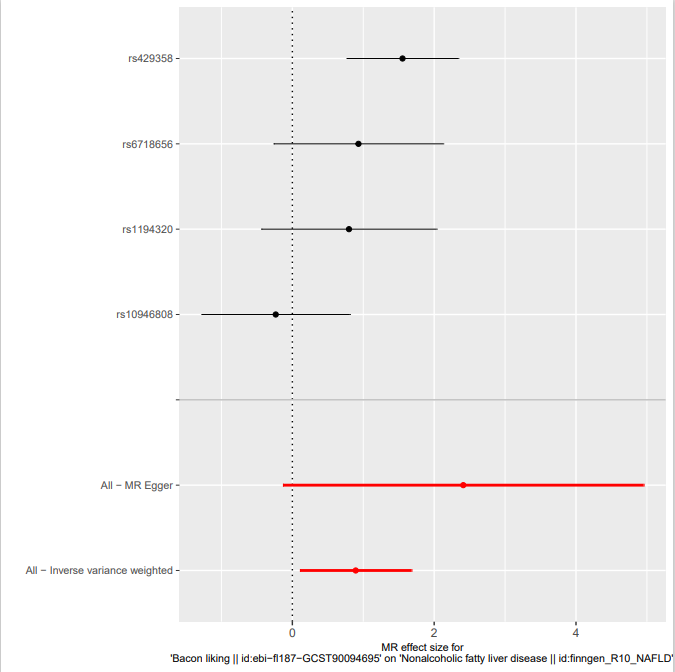

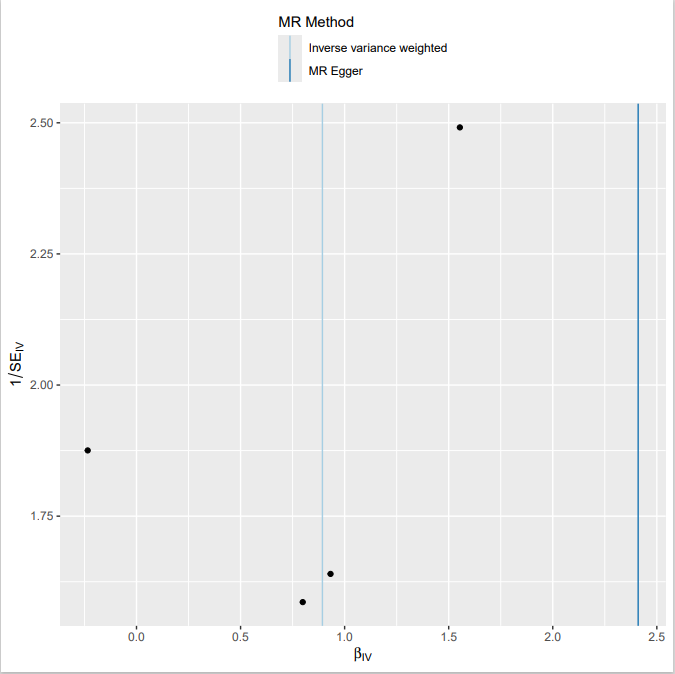


**Figure S11** Causal relationship between orange juice liking and NAFLD


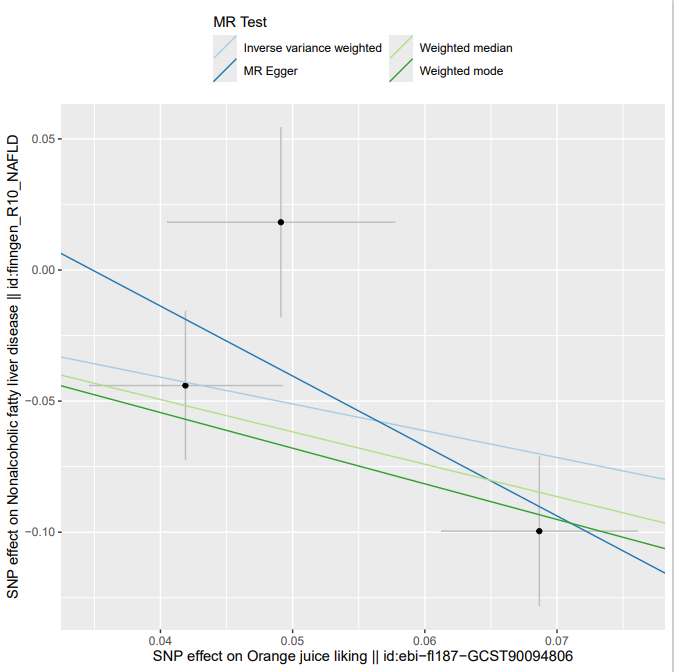

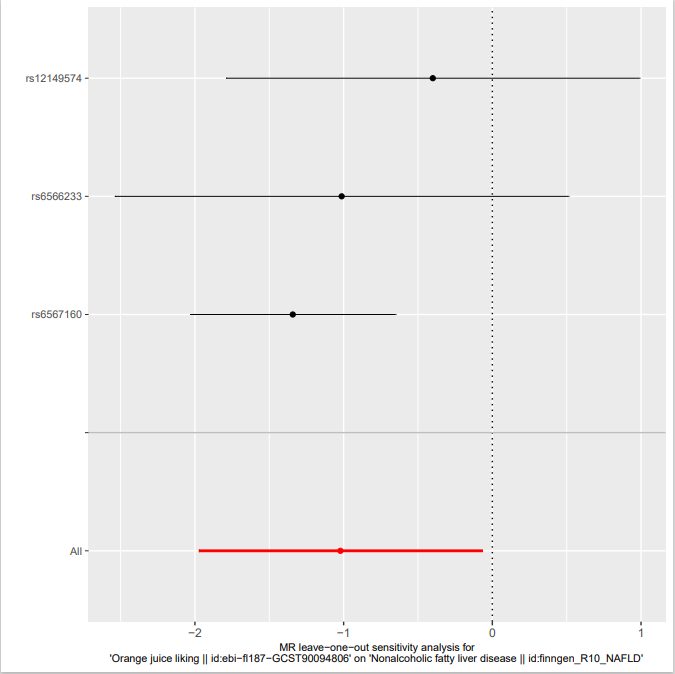


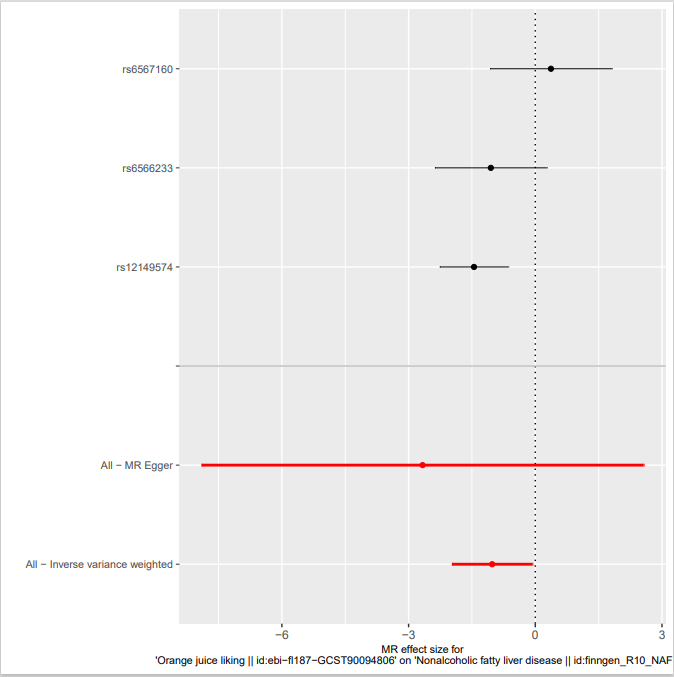

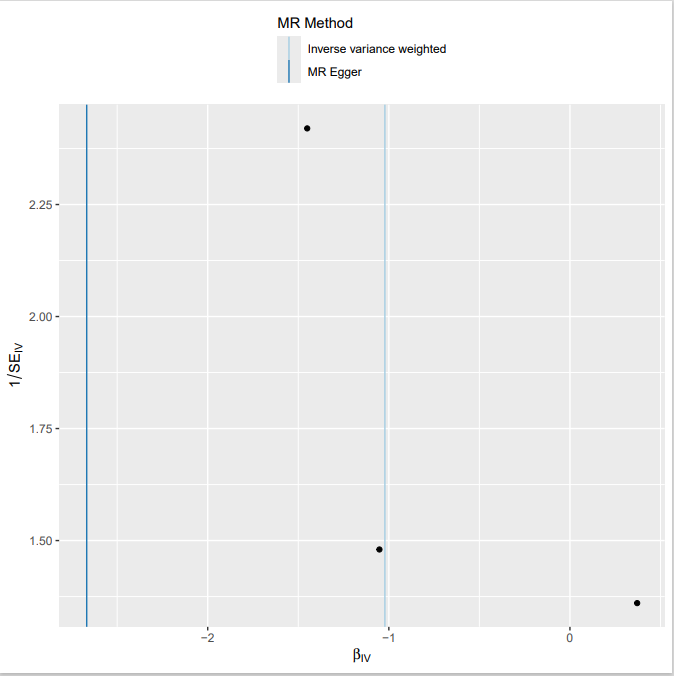


**Figure S12** Causal relationship between dried fruit liking and NAFLD


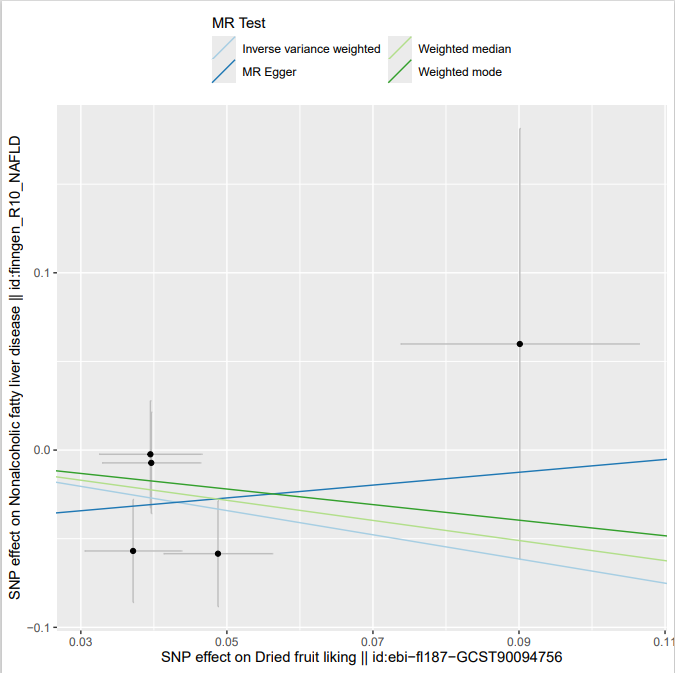

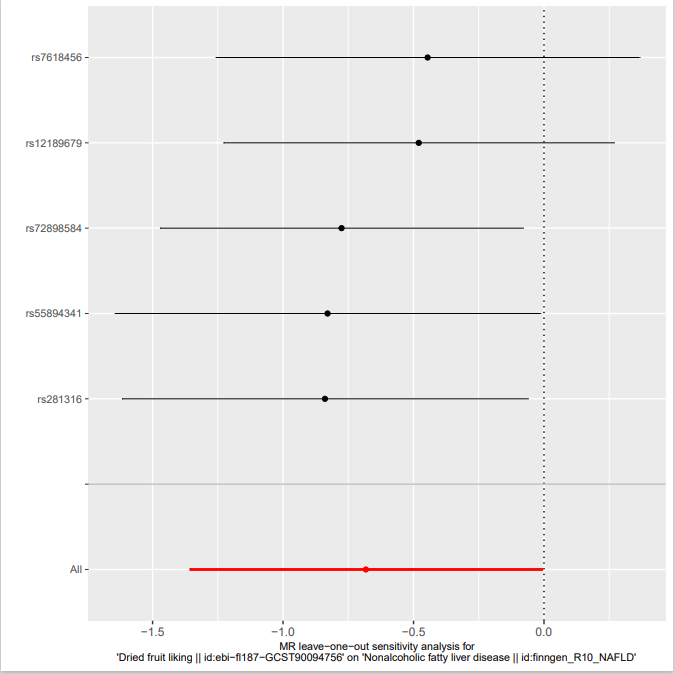


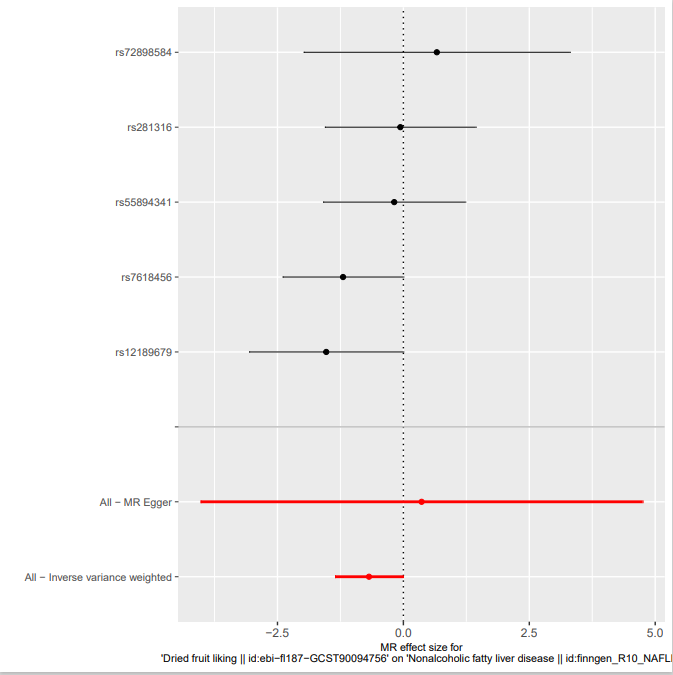

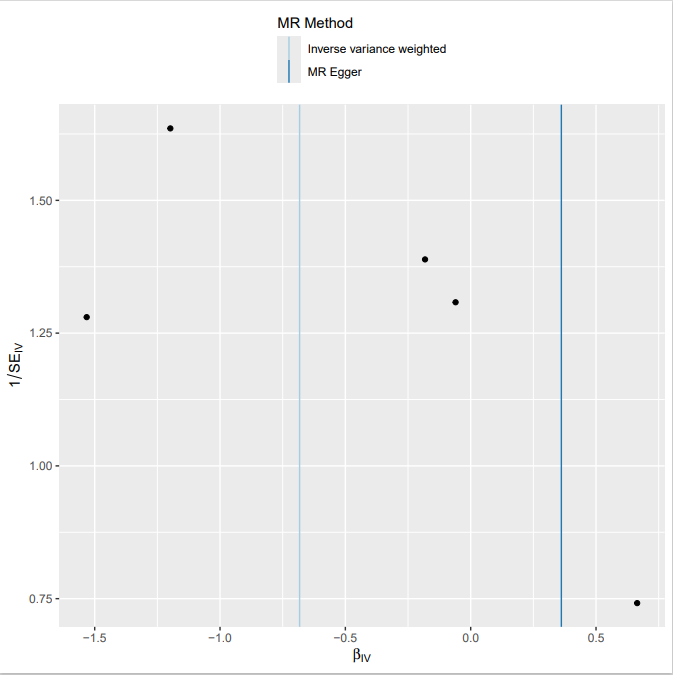

Supplement: Supplementary file 1 — Figure S1. [file FSN3-13-e70446-s004.docx]
